# Early Prediction of ICU Mortality in Patients with Acute Hypoxemic Respiratory Failure Using Machine Learning: The MEMORIAL Study

**DOI:** 10.3390/jcm14051711

**Published:** 2025-03-04

**Authors:** Jesús Villar, Jesús M. González-Martín, Cristina Fernández, José M. Añón, Carlos Ferrando, Juan M. Mora-Ordoñez, Domingo Martínez, Fernando Mosteiro, Alfonso Ambrós, Lorena Fernández, Isabel Murcia, Anxela Vidal, David Pestaña, Miguel A. Romera, Raquel Montiel, Ana M. Domínguez-Berrot, Juan A. Soler, Estrella Gómez-Bentolila, Ewout W. Steyerberg, Tamas Szakmany

**Affiliations:** 1 CIBER de Enfermedades Respiratorias, Instituto de Salud Carlos III, 28029 Madrid, Spain; josu.estadistica@gmail.com (J.M.G.-M.); jmaelizalde@gmail.com (J.M.A.); cafeoranestesia@gmail.com (C.F.); 2 Research Unit, Hospital Universitario Dr. Negrín, Fundación Canaria Instituto de Investigación Sanitaria, 35019 Las Palmas de Gran Canaria, Spain; 3 Li Ka Shing Knowledge Institute, St. Michael’s Hospital, Toronto, ON M5B 1W8, Canada; 4 Faculty of Health Sciences, Universidad del Atlántico Medio, 35017 Las Palmas, Spain; 5 Research Unit, Hospital Universitario Dr. Negrín, 35019 Las Palmas de Gran Canaria, Spain; cristina.fersan76@gmail.com (C.F.); estrellagbentolila@gmail.com (E.G.-B.); 6 Intensive Care Unit, Hospital Universitario La Paz, IdiPaz, 28029 Madrid, Spain; 7 Surgical Intensive Care Unit, Department of Anesthesia, Hospital Clinic, IDIBAPS, 08036 Barcelona, Spain; 8 Intensive Care Unit, Hospital Universitario Reginal Carlos Haya, 29010 Málaga, Spain; jumouci@gmail.com; 9 Intensive Care Unit, Hospital Universitario Virgen de la Arrixaca, 30120 Murcia, Spain; dmbct1@gmail.com (D.M.); juasobar@hotmail.com (J.A.S.); 10 Intensive Care Unit, Hospital Universitario A Coruña, 15006 La Coruña, Spain; fernando.mosteiro.pereira@sergas.es; 11 Intensive Care Unit, Hospital General Universitario de Ciudad Real, 13005 Ciudad Real, Spain; alfonsoa@sescam.jccm.es; 12 Intensive Care Unit, Hospital Universitario Río Hortega, 47012 Valladolid, Spain; mlfernandezrod@saludcastillayleon.es; 13 Intensive Care Unit, Complejo Hospitalario Universitario de Albacete, 02006 Albacete, Spain; murciasaez.im@gmail.com; 14 Intensive Care Unit, Hospital Universitario Fundación Jiménez Díaz, 28040 Madrid, Spain; anxelavidal@gmail.com; 15 Surgical Intensive Care Unit, Hospital Universitario Ramón y Cajal, 28034 Madrid, Spain; dpestanalag@hotmail.com; 16 Intensive Care Unit, Hospital Universitario Puerta de Hierro, 28222 Madrid, Spain; miguelangel.romera@salud.madrid.org; 17 Intensive Care Unit, Hospital Universitario N.S. de Candelaria, 38010 Santa Cruz de Tenerife, Spain; raquelmontiel@gmail.com; 18 Intensive Care Unit, Complejo Asistencial Universitario de León, 24008 León, Spain; adominguezb@saludcastillayleon.es; 19 Department of Biomedical Data Sciences, Leiden University Medical Center, 2333 ZA Leiden, The Netherlands; e.w.steyerberg@umcutrecht.nl; 20 Julius Center, University Medical Center Utrecht, 3584 CX Utrecht, The Netherlands; 21 Department of Anesthesia, Intensive Care and Pain Medicine, Cardiff University, Cardiff CF10 3AT, UK; szakmanyt1@cardiff.ac.uk

**Keywords:** acute hypoxemic respiratory failure, ICU mortality, clinical trials, lung-protective ventilation, machine learning, mortality prediction, observational studies

## Abstract

**Background**: Early prediction of ICU death in acute hypoxemic respiratory failure (AHRF) could inform clinicians for targeting therapies to reduce harm and increase survival. We sought to determine clinical modifiable and non-modifiable features during the first 24 h of AHRF associated with ICU death. **Methods**: This is a development, testing, and validation study using data from a prospective, multicenter, nation-based, observational cohort of 1241 patients with AHRF (defined as PaO_2_/FiO_2_ ≤ 300 mmHg on mechanical ventilation [MV] with positive end-expiratory pressure [PEEP] ≥ 5 cmH_2_O and FiO_2_ ≥ 0.3) from any etiology. Using relevant features captured at AHRF diagnosis and within 24 h, we developed a logistic regression model following variable selection by genetic algorithm and machine learning (ML) approaches. **Results**: We analyzed 1193 patients, after excluding 48 patients with no data at 24 h after AHRF diagnosis. Using repeated random sampling, we selected 75% (n = 900) for model development and testing, and 25% (n = 293) for final validation. Risk modeling identified six major predictors of ICU death, including patient’s age, and values at 24 h of PEEP, FiO_2_, plateau pressure, tidal volume, and number of extrapulmonary organ failures. Performance with ML methods was similar to logistic regression and achieved a high area under the receiver operating characteristic curve (AUROC) of 0.88, 95%CI 0.86–0.90. Validation confirmed adequate model performance (AUROC 0.83, 95%CI 0.78–0.88). **Conclusions**: ML and traditional methods led to an encouraging model to predict ICU death in ventilated AHRF as early as 24 h after diagnosis. More research is needed to identify modifiable factors to prevent ICU deaths.

## 1. Introduction

Acute hypoxemic respiratory failure (AHRF), as defined by PaO_2_/FiO_2_ ≤ 300 mmHg on positive end-expiratory pressure (PEEP) ≥ 5 cmH_2_O and FiO_2_ ≥ 0.3 under mechanical ventilation (MV), is a frequent and heterogeneous clinical syndrome in the intensive care unit (ICU) with a reported mortality ranging between 35% and 55% [1,2,3,4,5,6]. There is a wide variability in the definition and description of the baseline characteristics of this syndrome [1,5,6,7]. With a wide range of etiologies and manifestations (coma, acute heart failure, stroke, sepsis, pneumonia, trauma, etc.), it usually requires endotracheal intubation and MV. It is estimated that worldwide, 1 million patients develop AHRF every year [8]. Conversely, hypoxemia is common in patients on MV, although there is conflicting information regarding prevalence and outcome [1,2,3,4,5,6,7,8,9].

ICU patients have a broad range of baseline clinical characteristics that will progress for benefit or harm, despite the implementation of best management guidelines [10,11]. In general, critical care physicians have a limited ability to predict the death of AHRF patients in ICU very early, despite existing prediction models combining multiple variables driving prognosis [12,13]. Commonly used general risk prediction scores such as SAPS-II and APACHE-2 have shown to be unreliable [14]. A more accurate and clinically relevant estimation for assessment of ICU outcome might be beneficial for targeting therapeutic interventions in AHRF patients to avoid iatrogenic harm and to enhance organ dysfunction recovery. AHRF outcome is usually influenced by a wide spectrum of clinical features dependent and independent of pulmonary function [1,2,10,11,15,16]. Identifying modifiable clinical variables that could be associated with death in ICU within 24 h of therapy could suggest treatment alternatives to increase survival. The modern use of machine learning (ML), a healthcare innovation that identifies a recognizable problem with a likely solvable solution, could capture a complex interaction among variables [17,18] associated with AHRF outcome.

Few studies have investigated the prediction of ICU mortality in AHRF in the era of lung-protective MV. Predicting AHRF outcome could inform clinicians’ decision making by targeting specific therapeutic interventions to facilitate organ recovery, reduce harm, and decrease mortality. Therefore, in this study we aimed to assess the value of machine learning approaches in the development of a multivariable model for an early prediction of ICU death in patients with AHRF.

## 2. Methods

This is a secondary analysis of an observational, non-interventional, multicenter study, approved by the Ethics Committees of Hospital Universitario Dr. Negrín (Las Palmas de Gran Canaria, Spain, #2021-321-1), with preexisting ethical approval/exemptions allowing retrospective analysis [1]. The need for informed consent was waived based on Spanish legislation for biomedical research, due to the retrospective nature of analysis, anonymization/dissociation of data, and no potential harm or benefit to patients (Supplemental File). The study was conducted following the principles for medical research of the Declaration of Helsinki [19] and the transparent reporting of a multivariable prediction model for individual prognosis or diagnosis (TRIPOD) guidelines for prediction models [20].

We used a large dataset of AHRF patients representing the full diversity of AHRF patients seen in ICUs and treated with lung-protective MV [1]. We excluded patients who died or were extubated during the first day of AHRF diagnosis. All our patients were treated from admission into ICU. For prediction of ICU death, we followed three steps: (i) a methodology to report the model’s prediction, (ii) to present the performance of the prediction, and (iii) to explain the model’s reasoning.

### 2.1. Sites, Patient Populations and Study Design

We performed a comprehensive secondary analysis, termed the MEMORIAL (MachinE learning Model to predict ICU Outcome in patients with acute hypoxemic RespIratory fAiLure) Study, of an unrestricted dataset derived from 1241 adult (≥18 years) patients with AHRF [1] from any etiology, treated with lung-protective MV, conducted at 22 ICUs from 14 geographical areas of Spain, and enrolled during three periods covering several seasons (Supplemental File). Based on previous work [21], we focused our analysis on variables collected within the first 24 h of AHRF diagnosis to estimate early probability of ICU death, independent of any underlying disease or cause of death (Figure S1). We analyzed a total of 1193 patients (Table 1 and Table S1), after excluding 48 patients with no data at 24 h. Patients were excluded if they were extubated or died during the first day of AHRF diagnosis (Table S2). The unit of observation was having data collected at AHRF diagnosis (T0) and at 24 h (T24) (Table 1, Tables S1 and S2).

We used harmonized data from 22 hospitals across Spain (Supplemental File). We used variables (Tables S3 and S4) including demographics, comorbidities, cause of AHRF (or reason for MV), acute physiology and chronic health evaluation II (APACHE II) score [22] during the first 24 h of AHRF diagnosis, and data from ventilator settings and lung mechanics [tidal volume (VT), respiratory rate (RR), positive end-expiratory pressure (PEEP), plateau pressure (Pplat)], and gas exchange [(PaO_2_, PaCO_2_, FiO_2_, PaO_2_/FiO_2_, pH)] at T0 and T24. We recorded the sequential organ failure assessment (SOFA) score [23] and occurrence of extrapulmonary organ system failures (OFs) included in the SOFA scale at diagnosis of AHRF and 24 h later. Sepsis was defined by Sepsis-3 criteria [24]. We recorded the date and status (alive or dead) of patients at ICU and hospital discharge. Primary outcome was all-cause ICU mortality (defined as death while admitted into ICU).

### 2.2. Predefined Rules, Variable Selection, and Statistical Analysis

The study was conducted in three steps (Figure S2). For the first step (model training and testing) and third step (validation) we used random sampling for selecting 75.4% (n = 900) and 24.6% (n = 293), respectively (Supplemental File). We searched in the data for model specification since the model was not pre-specified. Once risk features were identified by univariate logistic regression analysis (Table S5), we performed a multivariable logistic regression analysis. In the second step, and since prediction models often perform poorly when assessed in validation studies, we performed internal–external validation by leaving out patients enrolled in each of the three periods (phases) once [1,25,26]. The strength for assessing internal–external validation increases when studies include patients from different hospitals, as in our patient population. We revalidated the model by testing it on 293 unseen patients.

Although we collected 246 variables in each patient during their ICU stay, variable selection has vital importance in developing an actionable and interpretable prediction model in clinical practice. Our goal for variable selection was to include clinically relevant features, avoiding redundant variables. We analyzed the following variables as potential predictors of ICU death: age at ICU admission, gender, comorbidities (only those with a prevalence ≥ 5%) (Tables S3 and S4), number of extrapulmonary OFs, SOFA score, PaO_2_, PaO_2_/FiO_2_, FiO_2_, PaCO_2_, pH, VT, RR, PEEP, Pplat, driving pressure (calculated as Pplat minus PEEP), and minute ventilation at T0 and T24 (Tables S6 and S7). No information on medication or special procedures was used in our prediction model. We defined and specified the statistical analysis plan before the final statistical analyses were conducted (Supplemental File).

We first performed descriptive statistical analyses. We performed a univariate analysis to predict ICU outcome and identified variables that could be included in the potential prediction model based on predefined rules and area under the receiver operating characteristic curves (AUCROC). Because the inclusion of all available variables in ML can lead to complex models, we screened the collected variables using a genetic algorithm (GA) variable selection method [27] to achieve parsimony with a small subset of variables while excluding redundant variables [28]. We applied GA to optimize the selected variables by minimizing the Akaike and the Bayesian information criteria (AIC, BIC) [29]. We report the variance inflation factor as a measure of multicollinearity in regression logistic analysis. A two-sided *p*-value < 0.005 was considered for identification of prognostic variables to keep the false discovery rate below 5% [30].

We constructed the MEMORIAL prediction model by considering the minimum number of features selected by GA that provided a similar performance as an all-variables prediction model. We used a five-fold cross-validation to randomly split the 900-patient cohort into 720 patients for training and 180 for testing (see Supplemental File). We evaluated this minimum number features model using logistic regression and three supervised ML methods: multilayer perceptron (MLP), random forest (RF), and support vector machine (SVM) [31,32] (Supplement File), to assess the performance using AUROC of each ML. Calculations were conducted using R Core Team software 2024 (R version 4.4.2 (https://www.r-project.org (R Foundation for Statistical Computing, Vienna, Austria). We assessed calibration and discrimination in a validation cohort of 293 unseen random patients for validation [33,34] (Supplemental File).

**Figure 1:**
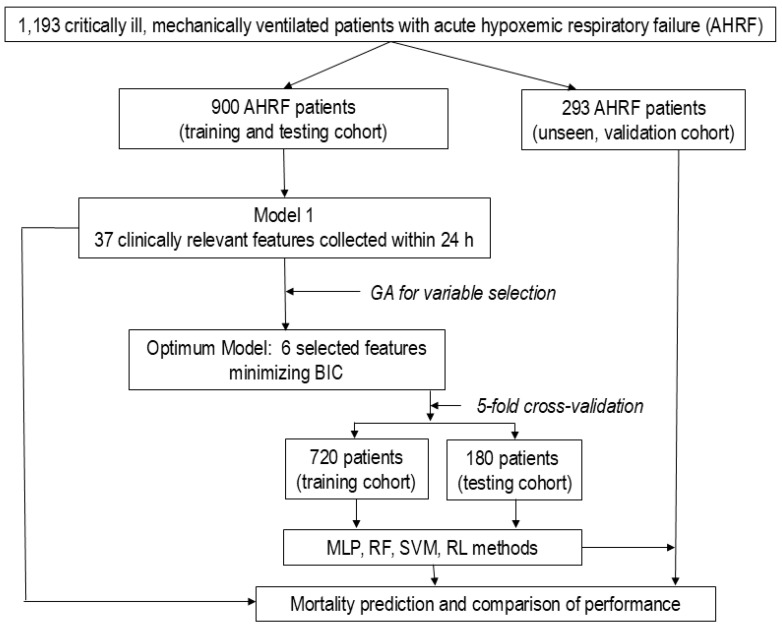
Diagram representing the study design. The flowchart illustrates the scheme for the database of 1193 patients with acute hypoxemic respiratory failure (AHRF), selection of variables for final analysis, machine learning approaches, and comparisons among prediction models. Once the most relevant variables were selected by a genetic algorithm (GA) in the dataset of 900 patients, this dataset was divided into five folders to perform five-fold randomized cross-validation, repeated 100 times using machine learning. AIC: Akaike information criterion, BIB: Bayesian information criterion, MLP: multilayer perceptron, RF: random forest, RL: logistic regression; SVM: support vector machine.

Figure 1 and Figure S2 summarize the study design.

## 3. Results

After removing 48 patients with no data at 24 h (Table S2), we included 1193 patients in our analysis (Table 1). ICU mortality was 35% (n = 416), with no differences in mortality (*p* = 0.686) among the parent (n = 1241), study (n = 1193), training/testing (n = 900), and validation cohorts (n = 293), respectively (Table 1 and Table 2). We observed a broad range of changes between values at baseline and after 24 h of routine ICU management. No patients were discharged and subsequently readmitted to the ICU during the study period. The median age at the time of AHRF was 65 years, with fewer women (32.4%) than men (67.6%). The patients’ race was not available in our datasets.

Only comorbidities with a >5% prevalence were considered in the model (Tables S3 and S4). In the univariate analysis, 16 variables had a prognostic relation with ICU death and 12 variables had an AUROC ≥ 0.60 (Table S5). In the multivariable logistic regression analysis, fewer features became predictors of ICU death (Tables S6–S10). The performance of the model with 37 variables had an AUCROC of 0.89 (95%CI 0.88–0.91), but most variables were correlated (Figures S3 and S4). After applying GA for variable selection using optimization of BIC, the AUCROC with six variables was 0.88 (95%CI 0.86–0.90) (Table 3) without strong multicollinearity (Table S10). Those six variables with strong relation to ICU death were age and values at T24 of PEEP, Pplat, FiO_2_, number of extrapulmonary OFs, and VT (Figure S5). The order of importance of those variables was: PEEP at T24, Pplat at T24, patient’s age at ICU admission, FiO_2_ at T24, number of extrapulmonary OFs at T24, and VT at T24. Of note, PEEP and VT at T24 had an OR < 1 and, therefore, were protective (greater values associated with lower mortality). A sensitivity analysis sustained these findings (Table S11). A data-driven stratification based on thresholds for those variables had a distinctive ICU mortality, mostly at T24.

Before implementing the ML model, we checked that patients and ICU deaths were similarly distributed in the three phases of the study (Tables S12–S14). Internal validation of the 6-variable model provided a high performance (AUCROC 0.88, 95%CI 0.84–0.93), using MLP or conventional logistic regression (Table S15). Internal–external validation by leaving each of the three phases out once provided an average AUCROC of 0.88 (95%CI 0.85–0.93) by MLP and 0.87 (95%CI 0.85–0.92) by logistic regression (Table S16). The validation cohort, using as few as 293 unseen patients, demonstrated a good performance of the model (AUCROC 0.83, 95%CI 0.78–0.88) (Table S17). Calibration and discrimination suggested good reliability of predictions, with logistic regression being as good as multiplayer perceptron ML (Figure S6).

## 4. Discussion

The main findings of this study are that prediction models of ICU mortality among patients with AHRF provided adequate performance whether developed by ML techniques or conventional regression analysis. Six clinical features (patient’s age, and values at T24 of PEEP, Pplat, FiO_2_, number of extrapulmonary OFs, and VT) contained the most prognostic information on ICU death within the first 24 h after diagnosis of AHRF. At the time that the epidemiological study was designed [1], the focus of lung-protective MV was to target VT, although recent data suggest that targeting driving pressure or mechanical power could be more effective in ventilated patients [35,36]. In our study, most patients with AHRF were ventilated according to the ARDS network and international societies’ criteria [37,38], whereas most patients in other studies did not receive proven or recommended approaches to lung-protective MV [2,9]. Clinical determinants of ICU death in AHRF are multifactorial. Apart from patient age, the rest of the five variables that were used to predict ICU mortality changed over 24 h with routine ICU management and treatment for each specific condition, although it is unknown whether combining precision medicine modalities for each predictor is synergistic. We are unaware of any complex interactions between the variables and treatment [11]. Even when complex interactions and treatment are identified among subgroups, a prediction model may unravel heterogeneity in treatment responses [10].

Previous observational studies in patients with acute respiratory failure had large variability in the definitions and description of baseline features, and had a lack of clinically relevant information on management and complications [2,3,4,7,39,40,41,42,43,44]. In our study, baseline characteristics were useless for predicting ICU outcome, and PaO_2_/FiO_2_ did not stratify patients by risk of death at baseline, but it worked at 24 h of AHRF diagnosis, independent of the use of Berlin criteria [45] or the 150-mmHg threshold [46]. Other studies have required the presence of parenchymal abnormalities, whereas our study did not mandate radiographic findings for diagnosis of AHRF. On the other hand, much of the information on differences in staffing, expertise, and practice surrounding managing AHRF with MV at individual hospitals may not be captured in the available clinical data [16]. Although there are a broad range of features that may modify the risk of ICU death, little is known about the true drivers of heterogeneity in treatment effects in AHRF (including patients and relative preferences, hospital load, and organization) [14,16]. We recognize that clinicians are often mostly interested in actionable and modifiable variables for improving expected outcomes [10].

Little is known about how many hospitals may actually be needed for robust training. Multicenter training allows the model to see more data and a more varied pattern of care, which may improve generalizability [47]. As data from more hospitals become available for training, models may become increasingly generalizable. Training and testing on data from several hospitals likely performs better compared with data trained only at a single hospital [48]. On the other hand, our sensitivity analysis assessed the robustness of our ML findings. We acknowledge that fundamental differences between hospitals or healthcare systems may affect the models’ ability to generalize to a given dataset, but we believe that our data adequately represent the range of clinical context encountered in ventilated AHRF patients. Changing a treating hospital may not always be an actionable intervention, although detailing current MV guidelines represents an important initial step for conducting further studies.

Based on our sensitivity analysis, it seems that most of the prognostic information in the first 24 h for predicting ICU death was due to the greatest changes occurring with adjustments of acute physiology, as suggested before [16]. One predictor is static (patient age) and the other five variables are modifiable, time-varying, from T0 (diagnosis of AHRF) to T24: applying PEEP, inspiratory Pplat, level of FiO_2_, treating extrapulmonary organ failure, and setting appropriate VT. It has been known that ICU outcome is worse with higher age [49], patients with severe hypoxemia requiring higher FiO_2_, and PEEP [13,46]. There is a direct relationship between Pplat and mortality [50], and the greater the number of extrapulmonary OFs, the higher the mortality [51]. To date, the best strategies to achieve improvement in AHRF have not been elucidated and should be subject to further research. Of note, PEEP at T24 and VT at T24 had an OR < 1 and, therefore, were protective (greater values were associated with lower mortality in AHRF), Predictors of ICU death were most relevant if collected close to 24 h, suggesting that ventilator, gas-exchange, and organ failure parameters at baseline are unhelpful for predicting outcome at the time of diagnosis of AHRF. We have previously shown that restricting AHRF severity to the hypoxemia level at baseline could lead to discrepancies in outcome prediction since hypoxemia is impacted by clinician-set ventilatory strategies [46].

The strengths of our study include the broad inclusion and limited exclusion criteria. Hence, our dataset represents the full diversity of AHRF patients seen in critical care units in most ICUs in the developed world. Second, the model identified six simple variables predicting ICU death that are routinely recorded and collected at the bedside of AHRF ventilated patients. Also, in our analysis, we were able to identify different subpopulations of AHRF patients with distinct mortality. Although one variable was static (patient age), the others changed with time, and contributed to high accuracy. Third, despite a similar protocol for this study, working very differently at participating hospitals, we assessed multicenter training and validation in the three phases and in the unseen cohort using tests across resampling folds. Fourth, we identified common variables that ICU clinicians have used in the management of AHRF across the world. Fifth, we think that a major finding of this study is that baseline features did not explain the individual’s likelihood of ICU outcome, suggesting that, in general, ICU treatment influences the potential outcome of AHRF patients.

We also acknowledge that this study has some limitations. First, the model does not include any information on medication, special procedures, or the socioeconomic status of patient population. Second, the model does not include information of staffing, hospital quality level, and individual and relative preferences, which are the main modifiable factors in recent publications [16]. Third, the study was conducted in a European country following international guidelines for the management of patients with AHRF [10,11,16,38]. Fourth, no patients with COVID-19 were enrolled, since the study was conducted in the pre-COVID era [1]. Finally, external validation of the proposed model in another clinical setting is needed to confirm performance before clinical implementation can be considered [25]. Any further validation of our model, including comparison to other general ICU risk predictions models, should be carried out in a new, prospectively collected dataset, preferably in a more diverse patient population.

In conclusion, six common variables are important to predict ICU mortality in ventilated patients with AHRF. Adherence to risk-precision-based management strategies may reduce the ICU mortality in AHRF patients. If the prediction model is further validated, clinicians, scientists, and health care administrators may impact the medical treatment that could improve the outcome of patients with AHRF.

## Figures and Tables

**Table 1:** Baseline characteristics and outcome data of 1241 ventilated patients with acute hypoxemic respiratory failure (AHRF) and 1193 patients with data at 24 h after AHRF diagnosis.

Variables	N = 1241 T0	N = 1193 T0	*p*-Value
Age, years, median (IQR)	65 (54–74)	65 (54–74)	
Age, years, mean ± SD	62.8 ± 14.3	62.7 ± 14.4	0.864
Sex	n (%: 95%CI)	n (%:95%CI)	
Male	834 (67.2: 64.6 to 69.8)	806 (67.6: 64.9 to 70.2)	0.888
Female	407 (32.8: 30.2 to 35.4)	387 (32.4: 29.8 to 35.1)	0.888
Etiology (reasons for invasive MV), n (%: 95%CI)			
Post-surgery	208 (16.8: 14.7 to 18.8)	190 (15.9: 13.9 to 18.0)	0.617
Stroke or coma	191 (15.4: 13.4 to 17.4)	189 (15.8: 13.8 to 17.9)	0.806
Pneumonia	169 (13.6: 11.7 to 15.5)	167 (14.0: 12.0 to 16.0)	0.823
Sepsis/Acute pancreatitis	152 (12.3;10.4 to 14.1)	146 (12.2: 10.4 to 14.1)	1
Trauma	151 (12.2: 10.4 to 14.0)	150 (12.6: 10.7 to 14.5)	0.806
Cardiac arrest	117 (9.4: 7.8 to 11.1)	108 (9.1: 7.4 to 10.7)	0.807
Cardiac failure/fluid overload	62 (5.0: 3.8 to 6.2)	59 (5.0: 3.7 to 6.2)	1
Aspiration/Inhalation	49 (4.0: 2.9 to 5.0)	47 (3.9: 2.8 to 5.0)	0.920
Others	137 (11.0: 9.3 to 12.8)	132(11.1: 9.3 to 12.8)	1
Unknown etiology	5 (0.4: 0 to 0.1)	5 (0.4: 0.0 to 0.1)	1
APACHE II score, mean ± SD	21.0 ± 8.0 ^§^	21.0 ± 7.8 ^§^	1.0
SOFA score, mean ± SD	8.95 ± 3.47	8.94 ± 3.39	0.943
FiO_2_, mean ± SD	0.63 ± 0.22	0.63 ± 0.21	1.0
PaO_2_, mmHg, mean ± SD	98.9 ± 34.6	98.8 ± 34.4	0.943
PaO_2_/FiO_2_, mmHg, mean ± SD	170.5 ± 64.1	170.9 ± 64.0	0.878
PaCO_2_, mmHg, mean ± SD	46.1 ± 12.4	45.9 ±12.0	0.686
pH, mean ± SD	7.32 ± 0.11	7.32 ± 0.11	1.0
VT, mL/kg PBW, mean ± SD	6.88 ± 1.07	6.89 ± 1.06	0.817
Respiratory rate, ventilator cycles/min, mean ± SD	19.7 ± 4.4	19.7 ± 4.4	1.0
Minute ventilation, L/min, mean ± SD	8.6 ± 2.1	8.6 ± 2.1	1.0
PEEP, cmH_2_O, mean ± SD	7.8 ± 2.8	7.8 ± 2.8	1.0
Plateau pressure, cmH_2_O, mean ± SD	22.3 ± 5.5	22.3 ± 5.4	1.0
Driving pressure, cmH_2_O, mean ± SD	14.5 ± 4.9	14.4 ± 4.8	0.611
No. extrapulmonary OFs, mean ± SD	1.72 ± 1.05	1.71 ± 1.03	0.813
Length of ICU stay, d, median (IQR)	10 (4–21)	11 (5–21)	0.449
Days from last day MV to ICU discharge, median (IQR)	2 (0–5)	2 (0–5)	0.734
All-cause ICU mortality, n (%: 95%CI)	438 (35.3: 32.6 to 38.0)	416 (34.9: 32.2 to 37.7)	0.862
All-cause hospital mortality, n (%: 95%CI)	514 (41.4: 38.7 to 44.2)	489 (41.0: 38.2 to 43.8)	0.862

APACHE: acute physiology and chronic health evaluation; AHRF: acute hypoxemic respiratory failure; CI: confidence intervals; d: days; FiO_2_: fraction of inspired oxygen concentration; ICU: intensive care unit; IQR: interquartile range; MV: mechanical ventilation; OF: organ failure; PBW: predicted body weight; PEEP: positive end-expiratory pressure; SD: standard deviation; SOFA: sequential organ failure assessment scale; T0: at AHRF diagnosis; VT: tidal volume. ^§^ APACHE II was not reported at baseline in 40 patients from the entire 1241 cohort, and in 39 from the 1193 patients.

**Table 2:** Baseline and outcome data of 1193 ventilated patients with acute hypoxemic respiratory failure, randomly sampled into training/testing cohort (n = 900) and validation cohort (n = 293).

Variables	N = 900 T0	N = 293 T0	*p*-Value
Age, years, median (IQR)	65 (54–74)	65 (55–74)	
Age, years, mean ± SD	62.5 ± 14.5	63.2 ± 13.9	0.469
Sex	n (%: 95%CI)	n (%:95%CI)	
Male	607 (67.4: 64.4 to 70.5)	199 (67.9: 62.6 to 73.3)	0.920
Female	293 (32.6: 29.5 to 35.6)	94 (32.1: 26.7 to 37.4)	0.920
Etiology (reasons for invasive MV), n (%: 95%CI)			
Post-surgery	136 (15.1: 12.8 to 17.5)	54 (18.4: 14.0 to 22.9)	0.209
Stroke or coma	140 (15.6: 13.2 to 17.9)	49 (16.7: 12.5 to 21.0)	0.699
Pneumonia	133 (14.8: 12.5 to 17.1)	34 (11.6: t.9 to 15.3)	0.206
Sepsis/Acute pancreatitis	113 (12.6: 10.4 to 14.7)	33 (11.3: 7.6 to 14.9)	0.632
Trauma	114 (12.7: 10.4 to 14.0)	36 (12.3: 8.5 to 16.1)	1.0
Cardiac arrest	79 (8.8: 6.9 to 10.6)	29 (9.9: 6.5 to 13.3)	0.647
Cardiac failure/fluid overload	44 (4.9: 3.5 to 6.3)	15 (5.1: 2.6 to 7.6)	1.0
Aspiration/Inhalation	37 (4.1: 2.8 to 5.4)	10 (3.4: 1.3 to 5.5)	0.718
Others	99 (11.0: 9.0 to 13.0)	33 (11.3: 7.6 to 14.9)	1.0
Unknown etiology	5 (0.6: 0 to 1)	0 (0: 0 to 0)	-
APACHE II score, mean ± SD	20.9 ± 7.9 ^§^	21.2 ± 7.7 ^§^	0.570
SOFA score, mean ± SD	8.9 ± 3.3	9.0 ± 3.5	0.657
FiO_2_, mean ± SD	0.63 ± 0.22	0.62 ± 0.21	0.495
PaO_2_, mmHg, mean ± SD	99.3 ± 35.6	97.5 ± 30.7	0.438
PaO_2_/FiO_2_, mmHg, mean ± SD	170.9 ± 63.8	170.8 ± 64.4	0.982
PaCO_2_, mmHg, mean ± SD	45.7 ± 12.0	46.7 ± 12.1	0.217
pH, mean ± SD	7.32 ± 0.11	7.31 ± 0.11	0.177
VT, mL/kg PBW, mean ± SD	6.9 ± 1.0	6.8 ± 1.1	0.147
Respiratory rate, ventilator cycles/min, mean ± SD	20 ± 4	20 ± 5	1.0
Minute ventilation, L/min, mean ± SD	8.6 ± 2.1	8.7 ± 2.1	0.479
PEEP, cmH_2_O, mean ± SD	8 ± 3	8 ± 3	1.0
Plateau pressure, cmH_2_O, mean ± SD	22 ± 5	22 ± 5	1.0
Driving pressure, cmH_2_O, mean ± SD	14 ± 5	14 ± 5	1.0
No. extrapulmonary OFs, mean ± SD	1.7 ± 1.0	1.8 ± 1.0	0.137
Length of ICU stay, d, median (IQR)	10 (7.22–21)	12 (5–21)	0.944
Days from last day MV to ICU discharge, median (IQR)	2 (0–5)	2 (0–6)	0.825
All-cause ICU mortality, n (%: 95%CI)	312 (34.7: 31.6 to 37.8)	104 (35.5: 30.0 to 41.0)	0.841
All-cause hospital mortality, n (%: 95%CI)	369 (41.0: 37.8 to 44.2)	120 (41.0: 35.3 to 46.6)	1.0

APACHE: acute physiology and chronic health evaluation; CI: confidence intervals; d: days; FiO_2_: fraction of inspired oxygen concentration; ICU: intensive care unit; IQR: interquartile range; MV: mechanical ventilation; OF: organ failure; PBW: predicted body weight; PEEP: positive end-expiratory pressure; SD: standard deviation; SOFA: sequential organ failure assessment scale; VT: tidal volume. ^§^ APACHE II was not reported at baseline in 30 patients from the entire 900 cohort, and in 9 from the 293 patients.

**Table 3:** Performance of a parsimonious model for predicting ICU mortality (6-variable model) within 24 h of diagnosis of AHRF using the genetic algorithm variable selection method, logistic regression analysis, and minimizing the Bayesian information criterion (BIC) in 900 patients. This model reduced the number of variables from 37 to 6. Data are expressed as mean values of logistic coefficients.

Variable	b	SE	OR	95% CI	*p*-Value
Intercept	−8.19	0.98	0	0–0	<0.001
Age	0.05	0.01	1.05	1.04–1.07	<0.001
VT at T24	−0.25	0.09	0.78	0.64–0.93	0.007
FIO_2_ at T24	1.78	0.64	5.92	1.71–20.79	0.005
PEEP at T24	−0.24	0.04	0.79	0.73–0.85	<0.001
Plateau pressure at T24	0.26	0.02	1.29	1.24–1.35	<0.001
No. extrapulmonary OFs at T24	0.87	0.1	2.38	1.97–2.89	<0.001
AIC	741.88				
BIC	775.4986				
AUC ROC	0.881 (0.860–0.903)				

AHRF: acute hypoxemic respiratory failure, AIC: Akaike information criterion, AUC ROC: area under the receiving operating characteristic curve, BIC: Bayesian information criterion, CI: confidence intervals, OF: extrapulmonary organ failures included in the sequential organ failure assessment scale, OR: odds ratio, SE: standard error, T24: at 24 h of diagnosis of AHRF, VT: tidal volume.

## Data Availability

All data needed to evaluate the conclusions in this article are presented and tabulated in the main text or the Supplemental File. Data are available from the corresponding author on reasonable request.

## Supplementary Materials

The following supporting information can be downloaded at: https://www.mdpi.com/article/10.3390/jcm14051711/s1. References [52,53,54,55,56,57,58,59,60,61,62,63,64,65,66,67,68] are cited in Supplementary Materials.

## References

[1] Villar J., Mora-Ordoñez J.M., Soler J.A., Mosteiro F., Vidal A., Ambrós A., Fernández L., Murcia I., Civantos B., Romera M.A. (2022). The PANDORA study: Prevalence and outcome of acute hypoxemic respiratory failure in the pre-COVID-19 era. Crit. Care Expl..

[2] Luhr O.R., Antonensen K., Karlsson M., Aardal S., Thorsteinsson A., Frostell C.G., Bonde J., The ARF Study Group (1999). Incidence and mortality after acute respiratory failure and acute respiratory distress syndrome in Sweden, Denmark, and Iceland. Am. J. Respir. Crit. Care Med..

[3] Linko R., Okkonen M., Pettilä V., Perttilä J., Parviainen I., Ruokonen E., Tenhunen J., Ala-Kokko T., Varpula T., FINNALI-Study Group (2009). Acute respiratory failure in intensive care units. FINNALI; a prospective cohort study. Intensive Care Med..

[4] Azevedo L.C., Park M., Salluh J.I., Rea-Neto A., Souza-Dantas V.C., Varaschin P., Oliveira M.C., Tierno P.F.G., dal-Pizzol F., Silva U.V. (2013). Clinical outcomes of patients requiring ventilatory support in Brazilian intensive care units: A multicenter, prospective, cohort study. Crit. Care.

[5] Wunsch H., Linde-Zwirbie W.T., Angus D.C., Hartman M.E., Milbrandt E.B., Kahn J.M. (2010). The epidemiology of mechanical ventilation use in the United States. Crit. Care Med..

[6] Kempker J.A., Abril M.K., Chen Y., Kramer M.R., Waller L.A., Martin G.S. (2020). The epidemiology of respiratory failure in the United States 2002-2017: A serial cross-sectional study. Crit. Care Expl..

[7] SRLF Trial Group (2018). Hypoxemia in the ICU: Prevalence, treatment, and outcome. Ann. Intensive Care.

[8] Mehta A.B., Syeda S.N., Wiener R.S., Walkey A.J. (2015). Epidemiological trends in invasive mechanical ventilation in the United States: A population-based study. J. Crit. Care.

[9] Bellani G., Laffey J.G., Pham T., Fan E., Brochard L., Esteban A., Gattinoni L., van Haren F., Larsson A., McAuley D.F. (2016). For the LUNG SAFE investigators and the ESICM trials group: Epidemiology, patterns of care, and mortality for patients with acute respiratory distress syndrome in intensive care units in 50 countries. JAMA.

[10] Maslove D.M., Tang B., Shankar-Hai M., Lawler P.R., Angus D.C., Baillie J.K., Baron R.M., Bauer M., Buchman T.G., Calfee C.S. (2022). Redefining critical illness. Nat. Med..

[11] Linck E.J.G., Goligher E.C., Semier M.W., Churpeck M.M. (2024). Toward precision in critical care research: Methods for observational and interventional studies. Crit. Care Med..

[12] Ferring M., Vincent J.L. (1997). Is outcome from ARDS related to the severity of respiratory failure?. Eur. Respir. J..

[13] Villar J., González-Martín J.M., Ambrós A., Mosteiro F., Martínez D., Fernández L., Soler J.A., Parra L., Solano R., Soro M. (2021). Stratification for identification of prognostic categories in the acute respiratory distress syndrome (SPIRES) score. Crit. Care Med..

[14] Xu C., Zheng L., Jiang Y., Jin L. (2023). A prediction model for predicting the risk of acute respiratory distress syndrome in sepsis patients: A retrospective cohort study. BMC Pulm. Med..

[15] Gajic O., Affessa B., Thompson B.T., Frutos-Vivar F., Malinchoc M., Rubenfeld G.D., Esteban A., Anzueto A., Hubmayr R.D. (2007). Prediction of death and prolonged mechanical ventilation in acute lung injury. Crit. Care.

[16] Churpeck M.M., Gupta S., Spicer A.B., Parker W.F., Fahrenbach J., Brenner S.K., Leaf D.E. (2021). Hospital-level variation in death for critically ill patients with COVID-19. Am. J. Respir. Crit. Care Med..

[17] Nemati S., Holder A., Razmi F., Stanley M.D., Clifford G.D., Buchman T.G. (2018). An interpretable machine learning model for accurate prediction of sepsis in the ICU. Crit. Care Med..

[18] Ding X.F., Li J.B., Liang H.Y., Wang Z.Y., Jiao T.T., Liu Z., Yi L., Bian W.S., Wang S.P., Zhu X. (2021). Predictive model for acute respiratory distress syndrome based on machine learning: A population-based study. Ann. Transl. Med..

[19] World Medical Association Declaration of Helsinki (2025). Ethical Principles for Medical Research Involving Human Participants. JAMA.

[20] Collins G.S., Reitsma J.B., Altman D.G., Mooms K.G.M. (2015). Transparent reporting of a multivariable prediction model for individual prognosis or diagnosis (TRIPOD): The TRIPOD statement. J. Clin. Epidemiol..

[21] Villar J., González-Martín J.M., Hernández-González J., Armengol M.A., Fernández C., Martín-Rodríguez C., Mosteiro F., Martínez D., Sánchez-Ballesteros J., Ferrando C. (2023). Predicting ICU mortality in acute respiratory distress syndrome patients using machine learning; the predicting outcome and stratification of severity in ARDS (POSTCARDS) study. Crit. Care Med..

[22] Knaus W.A., Draper E.A., Wagner D.P., Zimmerman J.E. (1985). APACHE II: A severity of disease classification system. Crit. Care Med..

[23] Vincent J.L., De Mendonça A., Cantraine F., Moreno R., Takala J., Suter P.M., Sprung C.L., Colardyn F., Blecher S. (1998). Use of the SOFA score to assess the incidence of organ dysfunction/failure in intensive care units: Results of a multicenter, prospective study. Working group on "sepsis-related problems" of the European Society of Intensive Care Medicine. Crit. Care Med..

[24] Singer M., Deutschman C.S., Seymour C.W., Shankar-Hari M., Annane D., Bauer M., Bellomo R., Bernard G.R., Chiche J.D., Coopersmith C.M. (2016). The third international consensus definitions for sepsis and septic shock (sepsis-3). JAMA.

[25] Steyerberg E.W., Harrel F.E. (2016). Prediction models need appropriate internal, internal-external, and external validation. J. Clin. Epidemiol..

[26] Steyerberg E.W., Harrel F.E., Borsboom G.J.J.M., Eijkemans M.J.C.R., Vergouwe Y., Habbema J.D.F. (2011). Internal validation of predictive models: Efficiency of some procedures for logistic regression analysis. J. Clin. Epidemiol..

[27] Scrucca L. (2013). GA: A package for genetic algorithms in R. J. Stat. Softw..

[28] González-Martín J.M., Sánchez-Medina A.J., Alonso J.B. (2019). Optimization of the prediction of financial problems in Spanish private health companies using genetic algorithm. Gac. Sanit..

[29] Vrieze S.I. (2012). Model selection and psychological theory: A discussion of the differences between the Akaike information criterion (AIC) and the Bayesian information criterion (BIC). Psychol. Methods.

[30] Ioannidis J.P.A. (2018). The proposal to lower P value threshold to 0.005. JAMA.

[31] Kim J.H., Kwon Y.S., Baek M.S. (2021). Machine learning models to predict 30-day mortality in mechanically ventilated patients. J. Clin. Med..

[32] Rashid M., Ramakrishnan M., Chandran V.P., Nandish S., Nair S., Shanbhag V., Thunga G. (2022). Artifical intelligence in acute respiratory distress syndrome: A systematic review. Artif. Intell. Med..

[33] Steyerberg E.W., Vergouwe Y. (2014). Towards better clinical prediction models: Seven steps for development and an ABCD for validation. Eur. Heart J..

[34] Van Calster B., Nieboer D., Vergouwe Y., De Cook B., Pencina M.J., Steyerberg E.W. (2016). A calibration hierarchy for risk models was defined: From utopia to empirical data. J. Clin. Epidemiol..

[35] Goligher E.C., Costa E.L., Yarnell C.J., Brochard L.J., Stewart T.E., Tomlinson G., Brower R.G., Slutsky A.S., Amato M.P. (2021). Effect of lowering Vt on mortality in acute respiratory distress syndrome varies with respiratory system elastance. Am. J. Respir. Crit. Care Med..

[36] Silva P.L., Ball L., Rocco P.R.M., Pelosi P. (2019). Power to mechanical power to minimize ventilator-induced lung injury?. Intensive Care Med. Exp..

[37] Acute Respiratory Distress Syndrome Network (2000). Ventilation with lower tidal volumes as compared with traditional tidal volumes for acute lung injury and the acute respiratory distress syndrome. N. Engl. J. Med..

[38] Fan E., Del Sorbo L., Goligher E.C., Hodgson C.L., Munshi L., Walkey A.J., Adhikari N.K., Amato M.B., Branson R., Brower R.G. (2017). An Official American Thoracic Society/European Society of Intensive Care Medicine/Society of Critical Care Medicine clinical practice guideline: Mechanical ventilation in adult patients with acute respiratory distress syndrome. Am. J. Respir. Crit. Care Med..

[39] Esteban A., Ferguson N.D., Meade M.O., Frutos-Vivar F., Apezteguia C., Brochard L., Raymondos K., Nin N., Hurtado J., Tomicic V. (2008). VENTILA Group: Evolution of mechanical ventilation in response to clinical research. Am. J. Respir. Crit. Care Med..

[40] Raymondos K., Dirks T., Quintel M., Molitoris U., Ahrens J., Dieck T., Johanning K., Henzler D., Rossaint R., Putensen C. (2017). Outcome of acute respiratory distress syndrome in university and non-university hospitals in Germany. Crit. Care.

[41] Caser E.B., Zandonade E., Pereira E., Gama A.M.C., Barbas C.S.V. (2014). Impact of distinct definitions of acute lung injury on its incidence and outcomes in Brazilian ICUs: Prospective evaluation of 7,133 patients. Crit. Care Med..

[42] Esteban A., Frutos-Vivar F., Muriel A., Ferguson N.D., Peñuelas O., Abraira V., Raymondos K., Rios F., Nin N., Apezteguía C. (2013). Evolution of mortality over time in patients receiving mechanical ventilation. Am. J. Respir. Crit. Care Med..

[43] Simonis F.D., Schouten L.R., Cremer O.L., Ong D.S., Amoruso G., Cinella G., Schultz M.J., Bos L.D. (2020). MARS consortium: Prognostic classification based on P/F and PEEP in invasively ventilated ICU patients with hypoxemia—insights from the MARS study. Intensive Care Med. Exp..

[44] Kopczynska M., Sharif B., Pugh R., Otahal I., Havalda P., Groblewski W., Lynch C., George D., Sutherland J., Pandey M. (2020). PANDORA-WALES investigators: Prevalence and outcomes of acute hypoxaemic respiratory failure in Wales: The PANDORA-WALES study. J. Clin. Med..

[45] Ranieri V.M., Rubenfeld G.D., Taylor Thompson B., Ferguson N.D., Caldwell E., Fan E., Camporota L., Slutsky A.S. (2012). Acute respiratory distress syndrome: The Berlin Definition. JAMA.

[46] Villar J., Fernández C., González-Martín J.M., Ferrando C., Añón J.M., Del Saz-Ortíz A.M., Díaz-Lamas A., Bueno-González A., Fernández L., Domínguez-Berrot A.M. (2022). Respiratory subsets in patients with moderate to severe acute respiratory distress syndrome for early prediction of death. J. Clin. Med..

[47] Wynants L., Kent D.M., Timmerman D., Lundquist C.M., van Calster B. (2019). Untapped potential of multicenter studies: A review of cardiovascular risk prediction model revealed inappropriate analysis and wide variation in reporting. Diagn. Progn. Res..

[48] Rockenschaub P., Hilbert A., Kossen T., Elbers P., von Dincklage F., Madai V.I., Frey D. (2024). The impact of multi-institution datasets on the generalizability of machine learning prediction models in the ICU. Crit. Care Med..

[49] Gee M.H., Gottlieb J.E., Albertine K.H., Kubis J.M., Peters S.P., Fish J.E. (1990). Physiology of aging related to outcome in the adult respiratory distress syndrome. J. Appl. Physiol..

[50] Shiu K.K., Rosen M.J. (2006). Is there a safe plateau pressure threshold for patients with acute lung injury and acute respiratory distress syndrome?. Am. J. Respir. Crit. Care.

[51] Aggarwal A.N., Agarwal R., Gupta D., Jindalet S.K. (2007). Nonpulmonary organ dysfunction and its impact on outcome in patients with acute respiratory failure. Chest.

[52] Parsa-Parsi R.W. (2022). The International Code of Medical Ethics of the World Medical Association. JAMA.

[53] Leisman D.E., Harhay M.O., Lederer D.J., Abramson M., Adjei A.A., Bakker J., Ballas Z.K., Barreiro E., Bell S.C., Bellomo R. (2020). Development and reporting of prediction models: Guidance for authors from editors of respiratory, sleep, and critical care journals. Crit. Care Med..

[54] Kacmarek R.M. (2019). Noninvasive respiratory support for postextubation respiratory failure. Respir. Care.

[55] Kacmarek R.M., Villar J., Sulemanji D., Montiel R., Ferrando C., Blanco J., Koh Y., Soler J.A., Martínez D., Hernández M. (2016). Open lung approach for the acute respiratory distress syndrome: A pilot, randomized controlled trial. Crit. Care Med..

[56] Eke G., Bloos F., Wilson D.C., Meybohm P., SepNet Critical Care Trials Group (2018). Identification of developing multiple organ failure in sepsis patients with low or moderate SOFA scores. Crit. Care.

[57] Villar J., Martínez D., Mosteiro F., Ambrós A., Añón J.M., Ferrando C., Soler J.A., Montiel R., Vidal A., Conesa-Cayuela L.A. (2018). Stratification and Outcome of Acute Respiratory Distress Syndrome (STANDARDS) Network. Crit. Care Med..

[58] Mandrekar J.N. (2010). Receiver operating characteristic curve in diagnostic test assessment. J. Thorac. Oncol..

[59] Rauf A., Sachdev A., Venkataraman S.T., Dinand V. (2021). Dynamic Airway Driving Pressure and Outcomes in Children With Acute Hypoxemic Respiratory Failure. Respir. Care.

[60] Kim J.H. (2019). Multicollinearity and misleading statistical results. Korean J. Anesthesiol..

[61] Wang X., Meng L., Zhang J., Zhao Z., Zou L., Jia Z., Han X., Zhao L., Song M., Zong J. (2023). Identification of ferroptosis-related molecular clusters and genes for diabetic osteoporosis based on the machine learning. Front. Endocrinol..

[62] Jolliffe I.T., Cadima J. (2016). Principal components analysis: A review and recent developments. Philos. Trans. A Math. Phys. Eng. Sci..

[63] Sayed M., Riaño D., Villar J. (2021). Predicting duration of mechanical ventilation in acute respiratory distress syndrome using supervised machine learning. J. Clin. Med..

[64] Boulesteix A.L., Janitza S., Kruppa J., König I.R. (2012). Overview of random forest methodology and practical guidance with emphasis on computational biology and bioinformatics. Wiley Interdiscip. Rev. Data Min. Knowl. Discov..

[65] Khalilzad Z., Hasasneh A., Tadj C. (2022). Newborn cry-based diagnostic system to distinguish between sepsis and respiratory distress syndrome using combined acoustic features. Diagnostics.

[66] Jeon E.T., Lee H.J., Park T.Y., Jin K.N., Ryu B., Lee H.W., Kim D.H. (2023). Machine learning-based prediction of in-ICU mortality in pneumonia patients. Sci. Rep..

[67] Saxena A., Mathur N., Pathak P., Tiwari P., Mathur S.K. (2023). Machine learning model based on insulin resistance metagenes underpins genetic basis of type 2 diabetes. Biomolecules.

[68] Martínez-Taboada F., Redondo J.I. (2020). The SIESTA (SEAAV Integrated evaluation sedation tool for anaesthesia) project: Initial development of a multifactorial sedation assessment tool for dogs. PLoS ONE.

