# Supplementary material for: Early Prediction of ICU Mortality in Patients with Acute Hypoxemic Respiratory Failure Using Machine Learning: The MEMORIAL Study
Source: J Clin Med. 2025 Mar 4;14(5):1711. doi: 10.3390/jcm14051711 (PMC11900058; doi:10.3390/jcm14051711)
Supplement: Supplementary file 1 [file jcm-14-01711-s001.zip › jcm-3487100-SI.pdf]

SUPPLEMENTAL FILE

This Supplemental File has been provided by the authors for additional information about their work

Early Prediction of ICU Mortality in Patients with Acute Hypoxemic Respiratory Failure using Machine Learning: The MEMORIAL Study

Jesús Villar, MD, PhD, FCCM*; **Jesús M. González-Martín**, PhD*; **Cristina Fernández**, MSc; **José M. Añón**, MD, PhD; **Carlos Ferrando**, MD, PhD; **Juan M. Mora-Ordoñez**, MD; **Domingo Martínez**, MD; **Fernando Mosteiro**, MD; **Alfonso Ambrós**, MD, PhD; **Lorena Fernández**, MD; **Isabel Murcia**, MD; **Anxela Vidal**, MD; **David Pestaña**, MD, PhD; **Miguel A. Romera**, MD; **Raquel Montiel**, MD; **Ana M. Domínguez-Berrot**, MD; **Juan A. Soler**, MD, PhD; **Estrella Gómez-Bentolila**, MSc; **Ewout W. Steyerberg**, PhD**; **Tamas Szakmany**, MD, PhD**

For the **M**achin**E** learning **M**odel to predict ICU **O**utcome in acute hypoxemic **R**esp**I**ratory f**A**i**L**ure (MEMORIAL) network

Corresponding author:
Jesús Villar, MD, PhD
Research Unit at Hospital Universitario Dr. Negrín, Fundación Canaria Instituto de Investigación Sanitaria de Canarias, Las Palmas de Gran Canaria. Barranco de la Ballena s/n, Annex building. 35019 Las Palmas de Gran Canaria, Spain.
Email: jesus.villar54@gmail.com

The MEMORIAL study

TABLE OF CONTENTS

ABBREVIATIONS page 3

SUPPLEMENTARY METHODS page 4

Ethical approval page 4

Patient population page 4

Outcome of interest page 6

General care page 6

Data collection and follow-up page 7

Statistical analysis plan page 9

- Predefined rules, pre-specified statistical analysis, and variable selection page 9
- Building the development and validation datasets page 11
- Validation page 12
- Data analysis page 12

SUPPLEMENTARY RESULTS page 14

- TABLES S1 to S17 pages 15–31
- FIGURES S1 to S6 pages 32–37

SUPPLEMENTARY REFERENCES page 38

APPENDIX S1. Centers and Members of the MEMORIAL Project page 42

ABBREVIATIONS

**AHRF**	Acute hypoxemic respiratory failure
**AIC**	Akaike information criterion
**APACHE**	Acute physiology and chronic health evaluation score
**AUC ROC**	area under the receiver operating characteristic curve
**BIC**	Bayesian information criterion
**CI**	Confidence interval
**COPD**	Chronic obstructive pulmonary disease
**FiO_2**	Fraction of inspired oxygen
**GA**	Genetic algorithm
**ICU**	Intensive care unit
**IQR**	Interquartile range
**ML**	Machine learning
**MLP**	Multilayer Perceptron
**MV**	Mechanical ventilation (invasive)
**NIV**	Non-invasive ventilation
**OF**	Organ failure
**OR**	Odds ratio
**MEMORIAL**	MachinE learning Model to predict intensive care unit Outcome in patients with acute hypoxemic RespIratory fAiLure
**PaO_2**	Partial pressure of oxygen in arterial blood
**PaO_2/FiO_2**	Partial pressure of oxygen in arterial blood to fraction of inspired oxygen ratio
**PBW**	Predicted body weight
**PEEP**	Positive end-expiratory pressure
**Pplat**	Plateau pressure
**RCT**	Randomized controlled trial
**RF**	Random forest
**RL**	Logistic Regression
**RM**	Recruitment maneuver
**RR**	Risk ratio
**SD**	Standard deviation
**SE**	Standard error
**SOFA**	Sequential organ failure assessment score
**SpO2**	Peripheral oxygen saturation
**SVM**	Support Vector Machine
**VFD**	Ventilator-free days
**VT**	Tidal volume
**T0**	At the time of diagnosis of AHRF
**T24**	24 h after diagnosis of AHRF

SUPPLEMENTARY METHODS

This comprehensive analysis, termed the MEMORIAL (**M**achin**E** learning **M**odel to predict ICU **O**utcome in patients with acute hypoxemic **R**esp**I**ratory f**A**i**L**ure) Study, was an investigator-initiated clinical study from a multicenter, non-interventional, observational study, conducted in a network of intensive care units (ICUs) from several geographical areas of Spain. MEMORIAL was registered at ClinicalTrial.gov on March 19, 2024 (NCT06333002).

Ethics Approval

This study was approved by the Ethics Committees of Hospital Universitario Dr. Negrín (Las Palmas de Gran Canaria, Spain) (#CEI/CEIm 2021-321-1). The requirement for informed consent was waived under the Royal Decrees 1090/2015 and 957/2020 based on the Spanish legislation for biomedical research due to the retrospective nature of this secondary analysis, the anonymization and dissociation of data, and no harm or benefit for managing patients.

We used an unrestricted dataset from our previously published study in patients with acute hypoxemic respiratory failure (AHRF) [1], after been approved by the Ethics Committees of Hospital Universitario La Paz, Madrid, Spain (#PI-2694) and Hospital Clínico Universitario de Valladolid, Spain (#PI17-594), and adopted by all participating centers. This study was conducted in accordance with the fundamental principles established in the Declaration of Helsinki, the Convention of the European Council related to human rights and biomedicine, the Ethical Guidelines for Health-related Research Involving Humans by the Council for International Organization of Medical Sciences of the World Medical Association [2], and within the requirements established by the Spanish legislation for biomedical research, the protection of personal data, and bioethics. None of the findings reported in the present study have been published elsewhere. The study followed the Transparent Reporting of a multivariable prediction model for Individual Prognosis or Diagnosis (TRIPOD) guidelines and recent guidelines for prediction models [3,4].

Patient population

We performed a comprehensive secondary analysis of an unrestricted set of data derived from 1241 adult (≥18 years) consecutive patients with AHRF [1] from any etiology, as defined by a $PaO_2/FiO_2 \leq 300$ mmHg on positive end-expiratory pressure (PEEP) ≥5 cmH_2O and $FiO_2 \geq 0.3$, treated with endotracheal intubation and lung-protective mechanical ventilation (MV) and conducted at 22 ICUs from 14 geographical areas of Spain, and enrolled during three periods or phases (1 May 2017 to 30

June 2017, 1 October 2017 to 30 November 2017, and 1 February 2018 to 31 March 2018), covering the four seasons to minimize seasonal effects and variability over time. Thus, our study applied only to adult patients receiving invasive MV with AHRF, although patients could have been on non-invasive respiratory support before intubation. In this study, the definition of AHRF did not require the presence of pulmonary or parenchymal abnormalities on chest imaging at the time of inclusion into the study, although chest imaging was mandatory according to the protocol for assessing pulmonary abnormalities at the day of study inclusion.

Based on previous work by our group [5], we focused our analysis on variables collected within the first 24 h of AHRF diagnosis to assess the early probability of ICU death, independent of underlying disease or cause of death (**Figure S1**). The unit of observation was to have collected data at the time of AHRF diagnosis (T0) and at 24 h (T24). T0 was defined as the day and time in which AHRF criteria were satisfied, irrespective of the date of ICU admission or initiation of MV. Patients were excluded if they were extubated or died during the first day of AHRF diagnosis. Thus, we excluded patients that were extubated, discharged, or died before the time of prediction (<24 h), and for which a clinician may be unlikely to use the model in practice. No patients were excluded (if they met all the inclusion criteria), regardless of age, sex, underlying disease, estimated life expectancy, or duration of invasive MV. All mechanically ventilated patients had arterial blood gases at study inclusion. We did not use SpO_2 as a surrogate for PaO_2 for enrolling patients.

We analyzed a total of 1193 patients (**Tables 1, S1**), after excluding 48 patients with no data at 24 h after AHRF diagnosis (**Table S2**). This study was conducted in three steps. In the first step we used random sampling for selecting 75.4% (n=900, as the cohort for model training and testing) and 24.6% (n=293, as the cohort for validation), respectively. We searched in the data for model specification since the model was not pre-specified. Once risk features were identified by univariate logistic regression analysis, we performed a multivariable logistic regression analysis. We used a 5-fold cross validation repeated 100 times to randomly split the 900-patient cohort into 720 for training and 180 for testing. In the second step, and since prediction models often perform poorly when assessed in validation studies, we performed internal–external validation by leaving out each of the three phases of our dataset once [1,6,7]. Final validation was conducted by testing the model in 293 unseen patients with a sufficient number of events (ICU deaths) required for validation [3,4]. The strength for assessing

internal–external validation increases when studies include patients from different hospitals, as in our patient population.

We used variables including demographics, comorbidities, cause of AHRF (or reason for MV), acute physiology and chronic health evaluation II (APACHE II) score [8] during the first 24 h of AHRF diagnosis, and data from ventilator settings and lung mechanics [tidal volume (VT), respiratory rate (RR), positive end-expiratory pressure (PEEP), plateau pressure (Pplat)], and gas exchange [(PaO_2, $PaCO_2$, FiO_2, PaO_2/FiO_2, pH)] at T0 and T24. We recorded the sequential organ failure assessment (SOFA) score [9] and occurrence of extrapulmonary organ system failures (OFs) included in the SOFA scale at diagnosis of AHRF and 24 h later. Sepsis was defined by Sepsis-3 criteria [10]. Clinical suspicion of infection was defined as co-occurrence of antibiotic administration and/or microbiological culture. We recorded the date and status (alive or dead) of patients at ICU and hospital discharge.

Outcome of interest

Primary outcome was all-cause ICU mortality. ICU mortality was defined as death while admitted into the ICU. All participating hospitals had ICU deaths in AHRF patients. We predicted the patients' risk of dying using patients with information from the first 24 hours (with data at T0 and at T24) of AHRF who were mechanically ventilated, alive, and in the ICU after 24 h of AHRF diagnosis.

General care

Although treatment was not strictly protocolized, attending clinicians followed current guidelines for general critical care management, which included the following: (i) in case of sepsis, physicians were urged to ensure early identification of causative microorganism, intravenous administration of antibiotics as soon as bacterial sepsis was suspected or recognized, and to optimize antibiotic selection and timely administration on the bases of antibiogram; (ii) fluid resuscitation and vasopressor use were individualized with the goal of maintaining a systolic blood pressure ≥90 mmHg or a mean arterial pressure ≥65 mmHg; and (iii) to maintain hemoglobin between 7-10 g/dL. For ventilatory management, clinicians followed current recommendations for lung-protective ventilation with a tidal volume (VT) of 4-8 mL/kg predicted body weight (PBW), a Pplat <30 cmH_2O, a ventilatory rate (RR) to maintain a $PaCO_2$ between 35-50 mmHg (permissive hypercapnia was allowed to target VT), and PEEP and FiO_2 combinations according to the PEEP-FiO_2 table of the ARDSnet protocol [11], ensuring that among the PEEP and FiO_2 combinations, clinicians should use the PEEP levels that allowed the reduction of FiO_2

to the lowest levels for maintaining a PaO_2 within a target range of 60 to 100 mmHg or to a SpO_2 within a target range of 90 to 98%.

The choice of drugs for sedation and analgesia, early neuromuscular blockade, prone positioning, hemodynamic management, and the decision to perform a tracheostomy were left to the discretion of attending physician. PBW was calculated using the following formula: 50 + 0.91 x [height (cm) – 152] for men, and 45.5 + 0.91 x [height (cm) – 152] for women [11]. Although prone positioning was used in some patients, we do not have data on the timing of prone positioning or whether prone ventilation was applied as a rescue therapy, as a routine practice, or following any specific protocol.

Weaning off MV could be started when the attending physician considered it clinically appropriate. Patients were assessed daily for readiness for a spontaneous breathing trials (SBTs) based on the ARDSnet protocol [11]. In general, prerequisites for the SBTs included a partial reversal of the underlying cause of AHRF, a PaO_2/FiO_2 >200 mmHg with PEEP<10 cmH_2O and FiO_2 ≤0.4, no vasopressors, continuous sedation minimized, and ability to cough during tracheal aspirations. Spontaneous ventilation was tested with a T-piece or with pressure support at 8 cmH_2O. The duration of the SBT was at least 30 min and no longer than 120 min. If the patient passed the SBT trial, a decision for extubation was taken, unless there was a specific reason not to extubate. Weaning and the decision to extubate were left to the discretion of the responsible physician. Since the rate of reintubation after extubation for all indications is estimated at about 20% [12], for patients at high risk for reintubation [>65 years of age, hypercapnic ($PaCO_2$ >45 mmHg after extubation), or ineffective cough and excessive secretions, with ≥1 weaning failure, with more than one comorbid condition, with upper airway obstruction, or with APACHE II score >12 on the day of extubation], non-invasive ventilatory support for 24 to 48 hours was indicated until they were stable or required reintubation [13].

Data collection and follow-up

Data were collected in each participating ICU using standardized case report forms (CRFs) and transmitted to the coordination center (Hospital Universitario Dr. Negrín, Las Palmas de Gran Canaria, Spain) when the patients was discharged from hospital. It is important to emphasize that for this high-quality dataset, the data manger, the coordinator, principal investigator, and all local investigators from participating ICUs attended a formal session in Madrid, Spain, for reviewing and discussing the study protocol and data collection on CRFs. All documents required for the study, including the study protocol, management guidelines, copies of CRFs, model of an informed consent form (in case it was needed)

were available for each attending clinician at each participating ICU, for ensuring compliance with the daily patient screening and inclusion into the study.

Before exporting the data into a computerized database, a trained data collector from the coordinating center checked the completeness and the quality of information. Logical checks were performed for missing data and for inconsistent findings, especially regarding clinical diagnosis, comorbidities, dates, and severity scores. No information on medication or special procedures were collected. If necessary, the data collector contacted the local investigator(s) to validate the data or reformat the data for entry into the database.

Onset of AHRF was defined as the day on which the patient first met all our inclusion criteria. For the purpose of this study, we collected data on clinical variables including demographics (age at ICU admission, sex, height, weight), main cause of AHRF, reason for intubation and MV, acute physiology and chronic health evaluation II (APACHE II) score [8] during the first 24 hours of AHRF diagnosis, physiological and laboratory results, management, hemodynamics (blood pressure and heart rate), data from ventilator settings and lung mechanics (VT, RR, PEEP, Pplat), and gas-exchange (PaO_2, $PaCO_2$, FiO_2, PaO_2/FiO_2, pH), at T0 and at T24. Attending physicians recorded comorbidities (arterial hypertension, neoplastic diseases, liver disease, cardiac disease, renal disease, immunosuppression, diabetes, obesity, pregnancy, chronic obstructive pulmonary disease, neurologic disease, neuromuscular disease, cerebrovascular disease, mental disorders, drug addiction, thyroid disease, organ transplantation, brain injury, coagulation diseases, etc.). Neoplastic disease included cancer in solid organs and hematological malignancies. Being immunosuppressed or immunocompromised was a result of certain diseases or conditions, or because of medication or treatment for a disease or condition, including but not limited to cancer or organ transplantation.

We recorded the occurrence of extrapulmonary OFs (cardiovascular system, liver, kidney, coagulation, and central nervous system) included in the SOFA scale at diagnosis of AHRF and after 24 h. Since the term "organ dysfunction" may emerge from reasons other than sepsis, extrapulmonary OF was defined as an acute change in organ-specific SOFA score ≥2 [10,14]. Baseline SOFA was assumed to be zero in patients without preexisting organ dysfunction. We recorded the duration of MV and the length of ICU and hospital stay. Also, we recorded the date and status (alive or dead) of patients at ICU and hospital discharge, and causes of death. Since overall mortality is a composite endpoint [15], we examined death from multiple causes (pulmonary (refractory hypoxemia, tension pneumothorax,

accidental ventilator disconnection), non-pulmonary (multiple system organ dysfunction, shock, terminal cancer, ventricular arrhythmias, brain death), limitation of therapeutic efforts for end-of-life, and others.

Statistical analysis plan

Predefined rules, pre-specified statistical analysis, and variable selection

We defined and specified rules and expectations in advance before the final statistical and machine learning (ML) analyses were conducted, realizing that overly detailed analyses could produce overoptimistic results due to a combination of reduced statistical power to detect real differences, an increase in the variance around the mean estimates, and/or an increased statistical likelihood of a false finding when many variables are examined. Therefore, variable selection is of vital importance in building a prediction model. Our aim for feature selection was to incorporate clinically relevant variables while avoiding noise/redundant variables.

First, for the purpose of our prediction model, we excluded comorbidities with a prevalence <5% (**Tables S3, S4**).

Second, we focused our analysis on clinically relevant variables collected within the first 24 hours of diagnosis of AHRF to estimate the early probability of ICU death, independent of the underlying disease or cause of death (**Figure S1**). As expected, the distribution of values for all variables identified a wide range of ICU mortality risk (**Table S5**). Although we recorded 246 variables during each patient's ICU stay, we analyzed the following variables as potential early predictors of ICU outcome: age at ICU admission, sex, comorbidities, SOFA score, number of extrapulmonary OFs, PaO_2, FiO_2, PaO_2/FiO_2 ratio, $PaCO_2$, pH, FiO_2, VT, RR, PEEP, Pplat, driving pressure (calculated as the difference between Pplat minus PEEP), and minute ventilation (as an indirect measurement of dead space, and calculated as VT x RR in liters/min) at the time of AHRF diagnosis and 24 hours later (**Table S5**). We did not include respiratory compliance in the model because it shares collinearity with three independent variables needed for its calculation (VT, Pplat, and PEEP) and it suffers from redundancy in the descriptive model. In addition, the respiratory compliance was found not to contribute to the predictive validity for mortality by a panel of experts [16]. Although we have the APACHE II score in most patients at T0 and T24, we did not include it in the model because it is a cumbersome score designed for the first 24 hours of ICU admission, made of twelve physiological variables and two disease-related variables; furthermore, it is not routinely calculated at the bedside in most ICUs worldwide or during trail enrollment decisions, it requires numerous data elements, and relies on laboratory data that are not uniformly collected. In

addition, at least half of the variables needed to calculate the APACHE II score are included in the list of selected variables, such as age, PaO_2, FiO_2, RR, pH, renal function, neurological function, and comorbidities.

Third, among the 37 clinically relevant variables which might influence AHRF outcome, we found that many variables reached statistical significance in relation to ICU mortality in the univariate analysis and/or had an area under the receiver (AUC ROC) ≥0.60, independently of the p-value (**Table S5**). An AUC ROC of 0.6 suggests a 60% chance that the clinician assessing the value of certain variable will correctly predict a survivor from a non-survivor at ICU discharge [17]. Several variables shared collinearity with other independent variables (for example, the calculation of PaO_2/FiO_2 with FiO_2 and PaO_2, Plat, and PEEP for the calculation of driving pressure), we considered all variables at the initial steps of analysis (**Tables S7, S8**). Whether driving pressure relates causally to outcome remains to be established in multicenter randomized controlled trials [18,19]. Other features seemed to have redundancy (**Tables S8, S9**, **Figure S3**).

Fourth, since the inclusion of all variables in a ML model can produce noisy results which are difficult to interpret, we screen variables using a genetic algorithm (GA) variable selection method [20] as a technique to achieve parsimony and identify a subset of relevant significant variables (subset selection) for a potential accurate prediction model while excluding noise/redundant variables. When applying GA for variable selection, we optimized the subset of selected variables by minimizing the Akaike information criterion (AIC) and Bayesian information criterion (BIC) [21]. AIC is an estimator of prediction error and thereby relative quality of statistical models for a given set of data. BIC is a criterion for model selection among a finite set of models. For both criteria, lower values are preferred.

Fifth, we also calculated the variance inflation factor (VIF), a measure of multicollinearity in regression logistic analysis. Multicollinearity exists when there is a correlation between multiple independent variables in a multiple regression model. To avoid multicollinearity, we built correlation matrices with data at T0 and at T24 as a statistical tool to calculate the linear relationship between two variables in the dataset [22,23] for excluding multicollinearity (**Figure S3**). The matrix shows how all possible pairs of values in a table are related to each other. It is a powerful tool for summarizing a large dataset and showing patterns in the data. The correlation coefficient ranges from -1 to +1, where 1 is considered a strong positive correlation between variables, 0 means a neutral relationship, and -1 means a negative correlation.

Sixth, we also performed a principal component analysis (PCA) as a statistical procedure that allows summarizing the information content by means of a smaller set of "summary indices" that can be more easily visualized and analyzed [24,25] (**Figure S4**). It is a popular multivariate statistical technique used in pattern recognition and signal processing based on projection methods. The goal of PCA is to extract the important information from the data and to express this information as a set of summary indices called principal components [24,25]. The first and second principal component represents the maximum variance in the data. The second principal component reflets the second largest source of variation in the data. When the two principal components are derived, they define a plane. In the specific context of our study, adding the numerical values of both components provides a tendency in the direction of data in favor of which data point seems better positioned to predict ICU death.

Seventh, we evaluated the final model with the minimum number of variables using conventional logistic regression and three supervised ML techniques: multilayer perceptron (MLP), random forest (RF) and support vector machine (SVM) [26-30]. The deep learning MLP is a feedforward neural network with a basic architecture comprising fully connected layers [29,30]. The input layer has the same number of inputs that the total predictor variables. The middle layer looks for characteristics associated with the data. The output layer had the same number of outputs as the categories to predict. RF is a supervised ML algorithm, which combines the output of multiple decision trees to reach a single result. The RF algorithm is made up of a collection of decision trees, and each tree in the ensemble is comprised of a data sample drawn from a training set with replacement [28]. While decision trees consider all the possible feature split, RF only selects a subset of those features, resulting in precise predictions. SVM is one of the prevailing algorithms because the data in biomedical research are often limited. One of the main strengths of the SVM is the ability to efficiently construct complex decision boundaries from limited samples [26,27].

In addition, we performed a sensitivity analysis (**Table S11**) to assess the robustness of our ML findings. In our sensitivity analysis, we examined the effects on how distinctive thresholds of the minimum variables of the prediction model would offer alternatives choices for improving survival, although we avoid assumptions on the estimation of treatment effects on those variables. Likely, although there is a reasonable degree of uncertainty attributable to modeling choices, the threshold associated with lower mortality would be the target for management.

Building the development (training and testing) and validation datasets

From the total of 1193 patients with data at T0 and T24, we used random sampling, and selected 75% (n=900) for model training and testing and 25% (n=293) for confirmation (validation).

In the *first step*, we described the 900 patients of the training/testing dataset. For ML techniques, input data were randomly split into training data (train), made up of 80% of the 900 records (720 rows), and the remaining 20% (180 rows) were used as testing data (**Figure 1**). This computerized technique replicates the process of sample generation by drawing samples with replacement from the original dataset [31], which means that the data set is divided into 5 folds, and in each run, 4 (80%) were used for training (n=720) and the remaining 1 (20%) was used for testing (n=180).

With the intention of reducing noise, each of the selected variables was normalized using the following formula: Z = X – min (X) / [max (X) – min (X)], where X is one of the selected variables, min (X) is the minimum value, max (X) is the maximum value, and Z is the resulting variable that was used for the ML process.

In the *second step*, and since prediction models often perform poorly when assessed in validation studies, we performed internal–external validation by leaving out patients enrolled in each of the three phases once [1,6,7]. Strength for assessing internal–external validation increases when studies include patients from different hospitals, as in our patient population.

Also, we compared the predictive performance of the three ML methods and logistic regression using the following parameters: accuracy, sensitivity, specificity, from the confusion matrix for both the validation and the test dataset [32] (**Tables S15, S16**). These values were calculated using the indicators true positive, false positive, false negative, and true negative.

Validation

In the *third step*, and for solving the complexity of validation of our prediction model, we tested the performance of the model in only 293 unseen patients with AHRF from multiple causes from all participating centers (**Table 2**). This cohort of unseen patients had a sufficient number of events (>100 ICU deaths) required for validation [4]. As recommended in guidelines, we avoided the retraining on validation dataset [4].

Data analysis

We calculated the mean, standard deviation (SD), median, and the interquartile range (IQR) of quantitative variables. We used the Kolmogorov–Smirnov test to examine the normal distribution of data. We calculated the frequency and percentage of qualitative variables. We reported data as percentages

or mean ± SD, unless otherwise specified. We reported the odds ratio and 95% confidence intervals (CI). We assessed differences in the values of clinically relevant features at T0 and T24, and across the development/testing cohort, internal–external validations cohorts, and validation cohort. We analyzed differences between distributions of categorical variables with the Fisher's exact test.

We identified potential variables that could be included in the prediction model based on our predefined rules and the AUC. For all comparisons, a two-sided significance level of p-value <0.005 was considered a real effect size, as recommended [33]. Since solely looking at a p-value threshold is an oversimplification, we acknowledge that p-values are inversely related to sample size (special consideration for the small dataset of the validation cohort) and that baseline characteristics at the time of AHRF diagnosis can be disrupted for the prediction model. Calculations were performed using the R Core Team software 2024 (R version 4.4.2) (https://www.r-project.org) (R Foundation for Statistical Computing, Vienna, Austria). We also calculated three measures (intercept, calibration slope, and *c*-statistic) to assess the validity of the prediction models, related to calibration and discrimination, and plotted graphically, by studying the validity of the models developed in 900 patients and validated in 293 patients [34,35].

SUPPLEMENTARY RESULTS

We used harmonized data from a total of 1193 patients in 22 hospitals (median 62 patients, 95%CI: 26-78), representing a total of 18,971 days of admission in the ICU (median 11 days, 95%CI: 5-21), although for the purpose of early prediction of ICU death, we focused on the variables collected within 24 hours of AHRF. The 900 patients of the development cohort stayed 14,382 days in the ICU [median 11 days, 95%CI: 5-20). The ICU mortalities in 1193 AHRF patients, in the development (training/testing) cohort, in the three phases of the study, and in the validation cohort were similar [416/1193 (34.9%), 312/900 (34.7%), 119/314 (37.9%), 91/273 (33.3), 102/313 (32.6%), 104/293 (35.5%), respectively (p=0.686) (**Tables 1, 2, S14**). The main comorbidities (prevalence >5%) in the development cohort (**Table S4**) were nine: arterial hypertension (52%), diabetes 27.6%), morbid obesity (21.8%), chronic obstructive pulmonary disease (15.3%), neoplastic disease (15.0), cardiac failure (14.6%), immunosuppressed (11.1%), chronic renal failure (8.0%), and chronic liver failure (5.2%). Sixteen percent of patients (n=144) had no comorbidities reported. However, no comorbidities were included in the final prediction model, after applying multivariate analysis and checking for performance using regression analysis and minimizing the Bayesian information criterion (**Table 3, Table S10**).

Only six variables had strong relation with early prediction of ICU death: patient's age on ICU admission, and values at T24 of PEEP, Pplat, FiO_2, number of extrapulmonary OFs and VT (**Table 3, Table S10**), suggesting that most features collected at baseline were irrelevant or useless for early prediction of ICU outcome in patients with AHRF. Therefore, models developed at T0 are not transferable at other periods. The order of importance of those variables was: (1) PEEP at T24, (2) Pplat at T24, (3) patient's age, (4) FiO_2 at T24, (5) number of extrapulmonary OFs at T24, and (6) VT at T24 (**Figure S5**). Of note, PEEP at T24 and VT at T24 had an OR<1 (**Table S8**) and, therefore, were protective (greater values of VT were associated with lower mortality in AHRF; lower values of PEEP were associated with higher mortality). A sensitivity analysis sustained these findings (**Table S11**). Distribution of patients based on thresholds for those variables had a distinctive ICU mortality. Categories for age, FiO_2, Pplat, and extrapulmonary OFs were associated with higher ICU mortality as the values of those features increased. Notably, Pplat>28 cmH_2O at T24 were associated with more than four times mortality in relation to patients with Pplat≤28 cmH_2O. It is likely that the use VT<4 ml/kg PBW and PEEP>12 cmH_2O at 24 h were in AHRF patients with most severe gas-exchange (as seen by thresholds of PaO_2/FiO_2, FiO_2, and PaO_2 values).

TABLE S1. Characteristics and outcome data of 1193 ventilated patients with data at baseline (T0) and at 24 h of diagnosis of acute hypoxemic respiratory failure (AHRF).

Variables	N=1193 T0	N=1193 T24	p-value
Age, years, median (IQR)	65 (54-74)	65 (54-74)	-
Age, years, mean ± SD	62.7 ± 14.4	62.7 ± 14.4	-
Sex, n (%: 95CI)			
Male	806 (67.56)	806 (67.56)	-
Female	387 (32.44)	387 (32.44)	-
Etiology, n (%: 95%CI)			1
Post-surgery	190 (15.93)	190 (15.93)	
Stroke or coma	189 (15.84)	189 (15.84)	
Pneumonia	167 (14.00)	167 (14.00)	
Sepsis/Acute pancreatitis	146 (12.24)	146 (12.24)	
Trauma	150 (12.57)	150 (12.57)	
Cardiac arrest	108 (9.05)	108 (9.05)	
Cardiac failure/fluid overload	59 (4.95)	59 (4.95)	
Aspiration/Inhalation	47 (3.94)	47 (3.94)	
Others	132 (11.06)	132 (11.06)	
Unknown etiology	5 (0.42)	5 (0.42)	
APACHE II score, mean ± SD	21.0 ± 7.8 §	19.2 ± 8.0 §	<0.001
SOFA score, mean ± SD	8.9 ± 3.4	8.4 ± 3.9	<0.001
FiO_2, mean ± SD	0.63 ± 0.21	0.54 ± 0.17	<0.001
PaO_2, mmHg, mean ± SD	98.8 ± 34.4	110.4 ± 34.0	<0.001
PaO_2/FiO_2, mmHg, mean ± SD	170.9 ± 64.0	231.2 ± 84.0	<0.001
$PaCO_2$, mmHg, mean ± SD	45.9 ±12.0	45.0 ± 9.8	0.045
pH, mean ± SD	7.32 ± 0.11	7.38 ± 0.09	<0.001
VT, mL/kg PBW, mean ± SD	6.89 ± 1.06	6.89 ± 1.02	1
Respiratory rate, ventilator cycles/min, mean ± SD	19.7 ± 4.4	21.8 ± 4.9	<0.001
Minute ventilation, L/min, mean ± SD	8.61 ± 2.06	9.51 ± 2.29	<0.001
PEEP, cmH_2O, mean ± SD	7.8 ± 2.8	8.6 ± 3.1	<0.001
Plateau pressure, cmH_2O, mean ± SD	22.3 ± 5.4	21.5 ± 5.7	<0.001
Driving pressure, cmH_2O, mean ± SD	14.4 ± 4.8	12.9 ± 5.0	<0.001
No. extrapulmonary OFs, mean ± SD	1.71 ± 1.03	1.70 ± 1.12	1
Length of ICU stay, d, median, IQR	11 (5 - 21)	11 (5 - 21)	-
Days from last day MV to ICU discharge, median, IQR	2 (0-5)	2 (0-5)	-
All-cause ICU mortality, n (%)	416 (34.9)	416 (34.9)	-
All-cause hospital mortality, n (%)	489 (41.0)	489 (41.0)	-

APACHE: acute physiology and chronic health evaluation; d: days; FiO_2: fraction of inspired oxygen concentration; ICU: intensive care unit; IQR: interquartile range; MV: mechanical ventilation; OF: organ failure; PBW: predicted body weight; PEEP: positive end-expiratory pressure; SD: standard deviation; SOFA: sequential organ failure assessment scale; VT: tidal volume.

§ APACHE II was not reported in 39 patients at baseline and at 24 h.

TABLE S2. Forty-eight excluded patients with acute hypoxemic respiratory (AHRF) with no data at 24 h. Causes of AHRF in 48 patients, and causes of death in 22 patients (45.8%) who died in the first day of AHRF.

Number of patients with no data at 24 h	N (%)	No. ICU deaths at ≤24 h of AHRF diagnosis (%) *
Etiology (reasons for invasive MV)	18 (37.5)	
Post-surgery	9 (18,8)	1 (4.5)
Cardiac arrest	6 (12.5)	8 (36.4)
Sepsis/acute pancreatitis	3 (6.3)	5 (22.7)
Cardiac failure/fluid overload	2 (4.2)	1 (4.5)
Pneumonia	2 (4,2)	2 (9.1)
Stroke or coma	2 (4,2)	2 (9.1)
Aspiration/inhalation	1 (2.1)	1 (4.5)
Trauma	5 (10.4)	1 (4.5)
Others		1 (4.5)
Total	48	22
Causes of death, n (%) *		
Multiple system organ failure	-	10 (45.4)
Irreversible shock	-	6 (27.3)
Brain death	-	3 (13.6)
Limitation of therapeutic efforts	-	2 (9.1)
Cardiac arrythmia	-	1 (4.6)

Abbreviations: (*) rate of death was calculated based on the total number of deaths (n=22); AHRF: acute hypoxemic respiratory failure; MV: mechanical ventilation; N: number of patients.

TABLE S3: Comorbidities in 1193 ventilated patients with acute hypoxemic respiratory failure (AHRF). *Note: Some patients could have more than one comorbidity.*

Comorbidities	Total (N=1193) No. (%) [95% CI]	ICU survivors (N=777) n (%) [95% CI]	ICU non-survivors (N=416) n (%) [95% CI]
Arterial hypertension	623 (52.2) [49.4 – 55.1]	375 (31.4) [28.8 – 34.1]	248 (20.8) [18.5 – 23.1]
Diabetes	330 (27.7) [25.1 – 30.2]	200 (16.8) [14.6 – 18.9]	130 (10.9) [9.1 – 12.7]
Morbid obesity	261 (21.9) [19.5 – 24.2]	170 (14.3) [12.3 – 16.2]	91 (7.6) [6.1 – 9.1]
COPD	191 (16.0) [13.9 – 18.1]	120 (10.1) [8.4 – 11.8]	71 (6.0) [4.6 – 7.3]
Cardiac failure	180 (15.1) [13.1 – 17.1]	105 (8.8) [7.2 – 10.4]	75 (6.3) [4.9 – 7.7]
Neoplastic diseases	170 (14.3) [12.3– 16.2]	89 (7.5) [6.0 – 9.0]	81 (6.8) [5.4 – 8.2]
Immunosuppressed	124 (10.4) [8.7 – 12.1]	63 (5.3) [4.0 – 6.6]	61 (5.1) [3.9 – 6.4]
Chronic renal disease	96 (8.1) [6.5 – 9.6]	50 (4.2) [3.1 – 5.3]	46 (3.9) [2.8 – 5.0]
Chronic liver failure	57 (4.8) [3.6 – 6.0]	31 (2.6) [1.7 – 3.5]	26 (2.2) [1.4 – 3.0]
Coronary artery disease	51 (4.3) [3.1 – 5.4]	34 (2.9) [1.9 – 3.8]	17 (1.4) [0. 8 – 2.1]
Other chronic pulmonary diseases	51 (4.3) [3.1 – 5.4]	32 (2.7) [1.8 – 3.6]	19 (1.6) [0. 9 – 2.3]
Non-invasive MV at home	42 (3.5) [2.5 – 4.6]	27 (2.3) [1.4 – 3.1]	15 (1.3) [0.6 – 1.9]
Neurological disease	34 (2.9) [1.9 – 3.8]	20 (1.7) [1.0 – 2.4]	14 (1.2) [0.6 – 1.8]
Mental disorders	32 (2.7) [1.8 – 3.6]	26 (2.2) [1.4 – 3.0]	6 (0.5) [0.1 – 0.9]
Thyroid disease	31 (2.6) [1.7 – 3.5]	19 (1.6) [0.9 – 2.3]	12 (1.0) [0.4 – 1.6]
Cerebrovascular disease	28 (2.4) [1.5 – 3.2]	15 (1.3) [0.6 – 1.9]	13 (1.1) [0.5 – 1.7]
Neuromuscular disease	26 (2.2) [1.4 – 3.0]	17 (1.4) [0.8 – 2.1]	9 (0.8) [0.3 – 1.3]
Organ transplantation	15 (1.3) [0.6 – 1.9]	1 (0.08) [-0.1 – 0.3]	14 (1.2) [0.6 – 1.8]
Others	34 (2.9) [1.9 – 3.8]	24 (2.0) [1.2 – 2.8]	10 (0.8) [0.3 – 1.4]
Patients with no comorbidities reported	198 (16.6) [14.5 – 18.7]	161 (13.5) [11.6 – 15.4]	37 (3.1) [2.1 – 4.1]

Note: Only the first **eight** comorbidities with ≥5% of patients (≥**60** patients) were considered for the prediction model. *Abbreviations: CI: confidence interval; COPD: chronic obstructive pulmonary disease; ICU: intensive care unit. MV: mechanical ventilation.*

Table S4: Comorbidities in 900 patients with acute respiratory failure. *Note: Some patients could have more than one comorbidity.*

Comorbidities	Total<br>No. (%) [95% CI]	ICU survivors<br>n (%) [95% CI]	ICU non-survivors<br>n (%) [95% CI]
Arterial hypertension	468 (52.0) [48.7 – 55.3]	286 (31.8) [28.7 – 34.8]	182 (20.2) [17.6 – 22.9]
Diabetes	248 (27.6) [24.6 – 30.5]	153 (17.0) [14.6 – 19.5]	95 (10.6) [8.6 – 12.6]
Morbid obesity	196 (21.8) [19.1 – 24.5]	131 (14.6) [12.3 – 16.9]	65 (7.2) [5.5 – 8.9]
COPD	138 (15.3) [13.0 – 17.7]	90 (10.0) [8.0 – 12.0]	48 (5.3) [3.9 – 6.8]
Neoplastic disease	135 (15.0) [12.7– 17.3]	69 (7.7) [5.9 – 9.4]	66 (7.3) [5.6 – 9.0]
Cardiac failure	131 (14.6) [12.3 – 16.9]	74 (8.2) [6.4 – 10.0]	57 (6.3) [4.7 – 7.9]
Immunosuppressed	100 (11.1) [9.1 – 13.2]	51 (5.7) [4.2 – 7.2]	49 (5.4) [4.0 – 7.0]
Chronic renal disease	72 (8.0) [6.2 – 9.8]	36 (4.0) [2.7 – 5.3]	36 (4.0) [2.7 – 5.3]
Chronic liver failure	47 (5.2) [3.8 – 6.7]	27 (3.0) [1.9 – 4.1]	20 (2.2) [1.3 – 3.2]
Coronary artery disease	39 (4.3) [3.0 – 5.7]	27 (3.0) [1.9 – 4.1]	12 (1.3) [0. 6 – 2.1]
Other chronic pulmonary diseases	40 (4.4) [3.1 – 5.8]	27 (3.0) [1.9 – 4.1]	13 (1.4) [0. 7 – 2.2]
Non-invasive mechanical ventilation at home	25 (2.8) [1.7 – 3.9]	18 (2.0) [1.1 – 2.9]	7 (0.8) [0.2 – 1.4]
Neurological disease	28 (3.1) [2.0 – 4.3]	18 (2.0) [1.1 – 2.9]	10 (1.1) [0.4 – 1.8]
Mental disorders	25 (2.8) [1.7 – 3.9]	22 (2.4) [1.4 – 3.5]	3 (0.3) [-0.0 – 0.7]
Thyroid disease	21 (2.3) [1.4 – 3.3]	11 (1.2) [0.5 – 1.9]	10 (1.1) [0.4 – 1.8]
Cerebrovascular disease	19 (2.1) [1.2 – 3.1]	9 (1.0) [0.4 – 1.7]	10 (1.1) [0.4 – 1.8]
Neuromuscular disease	21 (2.3) [1.4 – 3.3]	13 (1.4) [0.7 – 2.2]	8 (0.9) [0.3 – 1.5]
Organ transplantation	9 (1.0) [0.4 – 1.7]	0 (0.0) [0 – 0]	9 (1.0) [0.4 – 1.7]
Others	26 (2.9) [1.8 – 4.0]	19 (2.1) [1.2 – 3.1]	7 (0.8) [0.2 – 1.4]
Patients with no comorbidities reported	144 (16.0) [13.6 – 18.4]	115 (12.8) [10.6 – 15.0]	29 (3.2) [2.1 – 4.4]

Note: Only the first **nine** comorbidities with ≥5% of patients (≥**45** patients) were considered for the prediction model.

TABLE S5. Univariate logistic regression of clinically relevant variables in 900 patients with acute hypoxemic respiratory failure (AHRF).

Variables	N	Beta	SE	OR (95% CI)	p-value	AUC ROC (95% CI)
Age	900	0.03	0.01	1.04 (1.02-1.05)	<0.001	**0.63** (0.59-0.67)
Gender	900	0.08	0.15	1.08 (0.8-1.44)	0.609	0.51 (0.48-0.54)
Arterial hypertension	900	0.39	0.14	1.48 (1.12-1.95)	0.006	0.55 (0.51-0.58)
Cardiac failure	900	0.44	0.19	1.55 (1.06-2.26)	0.022	0.53 (0.5-0.55)
Chronic renal failure	900	0.69	0.25	2.0 (1.23-3.25)	0.005	0.53 (0.51-0.55)
Diabetes	900	0.22	0.15	1.24 (0.92-1.68)	0.157	0.52 (0.49-0.55)
Immunocompromised	900	0.67	0.21	1.96 (1.29-2.98)	0.002	0.54 (0.51-0.56)
Morbid obesity	900	-0.09	0.17	0.92 (0.65-1.28)	0.617	0.51 (0.48-0.54)
COPD	900	0.01	0.19	1.01 (0.68-1.47)	0.975	0.50 (0.48-0.53)
Neoplastic disease	900	0.7	0.19	2.02 (1.39-2.92)	<0.001	0.55 (0.52-0.57)
Chronic liver failure	900	0.35	0.3	1.42 (0.78-2.57)	0.245	0.51 (0.49-0.53)
SOFA at T0	900	0.2	0.02	1.22 (1.17-1.28)	<0.001	**0.68** (0.64-0.71)
SOFA at T24	900	0.28	0.02	1.32 (1.26-1.38)	<0.001	**0.76** (0.73-0.79)
VT at T0	900	-0.12	0.07	0.89 (0.78-1.01)	0.075	0.52 (0.48-0.56)
VT at T24	900	-0.16	0.07	0.86 (0.75-0.98)	0.024	0.53 (0.49-0.57)
FiO_2 at T0	900	0.74	0.32	2.1 (1.12-3.93)	0.021	0.55 (0.51-0.59)
FIO_2 at T24	900	2.88	0.42	17.89 (7.94-41.15)	<0.001	**0.63** (0.59-0.66)
Respiratory rate at T0	900	0.02	0.02	1.02 (0.98-1.05)	0.338	0.51 (0.47-0.55)
Respiratory rate at T24	900	0.06	0.01	1.06 (1.03-1.09)	<0.001	0.57 (0.53-0.61)
PEEP at T0	900	0.01	0.03	1.01 (0.96-1.06)	0.712	0.51 (0.47-0.55)
PEEP at T24	900	0.04	0.02	1.04 (1-1.09)	0.057	0.54 (0.50-0.58)
Plateau pressure at T0	900	0.14	0.01	1.15 (1.12-1.18)	<0.001	**0.69** (0.65-0.73)
Plateau pressure at T24	900	0.2	0.02	1.23 (1.19-1.26)	<0.001	**0.77** (0.74-0.81)
Driving pressure at T0	900	1.34	0.15	3.83 (2.87-5.13)	<0.001	**0.66** (0.63-0.69)
Driving pressure at T24	900	2.26	0.17	9.55 (6.84-13.47)	<0.001	**0.72** (0.69-0.75)
Minute ventilation at T0	900	-0.04	0.03	0.96 (0.9-1.03)	0.251	0.48 (0.44-0.52)
Minute ventilation at T24	900	0.05	0.03	1.05 (0.99-1.11)	0.134	0.53 (0.49-0.57)
PaO_2 at T0	900	0	0	1 (1-1)	0.751	0.51 (0.47-0.55)
PaO_2 at T24	900	0	0	1 (0.99-1)	0.244	0.54 (0.50-0.58)
PaO_2/FiO_2 at T0	900	0	0	1 (1-1)	0.239	0.53 (0.49-0.57)
PaO_2/FiO_2 at T24	900	0	0	1 (0.99-1)	<0.001	**0.60** (0.56-0.64)
$PaCO_2$ at T0	900	0	0.01	1 (0.99-1.02)	0.456	0.51 (0.46-0.55)
$PaCO_2$ at T24	900	0.02	0.01	1.02 (1-1.03)	0.016	0.52 (0.48-0.57)
pH at T0	900	-2.08	0.65	0.13 (0.03-0.45)	0.001	0.56 (0.52-0.60)
pH at T24	900	-6.61	0.89	0 (0-0.01)	<0.001	**0.63** (0.59-0.67)
No. extrapulmonary OFs at T0	900	0.61	0.07	1.84 (1.59-2.13)	<0.001	**0.66** (0.63-0.70)
No. extrapulmonary OFs T24	900	0.91	0.08	2.5 (2.14-2.94)	<0.001	**0.75** (0.71-0.78)

AHRF: acute hypoxemic respiratory failure, AUC ROC: area under the receiving operating characteristics curve; CI: confidence intervals; OF: organ failure; OR: odds ratio; PEEP: positive end-expiratory pressure; SE: standard error; SOFA: sequential organ failure assessment score; T0: at the time of AHRF diagnosis; T24: at 24 h after AHRF diagnosis; VT: tidal volume.

TABLE S6. Multivariate analysis and performance of a model of predicting ICU mortality considering all variables (n=37) within 24 hours of diagnosis of AHRF using logistic regression analysis. Data are expressed as mean values of logistic coefficients.

Variable	b	SE	OR	95% CI	p-value
(Intercept)	8.34	11.51	4169.92	0 – 3.04E+13	0.469
Age	0.05	0.01	1.06	1.04 - 1.08	<0.001
Gender (female)	0.31	0.34	1.36	0.7 - 2.63	0.362
Arterial hypertension	0.06	0.24	1.06	0.66 - 1.69	0.817
Cardiac failure	0.05	0.29	1.06	0.59 - 1.87	0.853
Chronic renal failure	0.24	0.36	1.27	0.63 - 2.55	0.506
Diabetes	0.29	0.23	1.34	0.85 - 2.1	0.207
Immunocompromised	0.41	0.34	1.5	0.78 - 2.9	0.224
Morbid obesity	0.19	0.26	1.21	0.72 - 2	0.470
COPD	-0.22	0.29	0.8	0.45 - 1.4	0.437
Neoplastic disease	0.54	0.27	1.72	1 - 2.96	0.048
Chronic liver failure	-0.43	0.46	0.65	0.26 - 1.58	0.346
SOFA at T0	-0.09	0.09	0.91	0.76 - 1.08	0.296
SOFA at T24	0.15	0.08	1.17	0.99 - 1.37	0.061
VT at TO	-0.32	0.35	0.72	0.36 - 1.44	0.361
VT at T24	-0.31	0.38	0.73	0.35 - 1.53	0.401
FiO_2 at TO	1.4	1.38	4.05	0.27 - 60.79	0.310
FIO_2 atT24	1.96	0.94	7.12	1.15 - 46.11	0.037
Respiratory rate at TO	-0.13	0.11	0.88	0.7 - 1.09	0.236
Respiratory rate at T24	-0.01	0.1	0.99	0.82 - 1.22	0.934
PEEP at TO	-0.08	0.06	0.92	0.81 - 1.04	0.176
PEEP at T24	-0.2	0.06	0.82	0.72 - 0.92	<0.001
Plateau pressure at TO	0.02	0.04	1.02	0.93 - 1.11	0.715
Plateau pressure at T24	0.28	0.04	1.32	1.21 - 1.44	<0.001
Driving pressure >15 at T0	-0.55	0.36	0.58	0.28 - 1.18	0.133
Driving pressure >15 at T24	0.31	0.38	1.37	0.65 - 2.9	0.414
Minute ventilation at T0	0.24	0.26	1.28	0.77 - 2.14	0.349
Minute ventilation at T24	0.05	0.24	1.05	0.65 - 1.66	0.841
PaO_2 at TO	-0.01	0.01	0.99	0.98 - 1.01	0.439
PaO_2 at T24	0	0	1	0.99 - 1.01	0.559
PaO_2/FiO_2 at TO	0.01	0.01	1.01	1 - 1.02	0.230
PaO_2/FiO_2 at T24	0	0	1	1 - 1.01	0.702
$PaCO_2$ at TO	0	0.01	1	0.97 - 1.02	0.816
$PaCO_2$ at T24	-0.02	0.01	0.98	0.95 - 1	0.099
pH at TO	1.71	1.29	5.53	0.45 - 71.21	0.184
pH at T24	-3.81	1.53	0.02	0 - 0.42	0.013
Number extrapulmonary OFs at T0	0.02	0.27	1.03	0.6 - 1.74	0.927
Number of extrapulmonary OFs at T24	0.58	0.26	1.79	1.07 - 3.02	0.028
AIC	758.25				
BIC	940.74				
AUC ROC	0.897 (0.877 – 0.918)				

AHRF: acute hypoxemic respiratory failure, AIC: Akaike information criterion, AUC ROC: area under the receiving operating characteristic curve, BIC: Bayesian information criterion, CI: confidence intervals, COPD: chronic obstructive pulmonary disease, OF: organ failures, OR: odds ratio, PEEP: positive end-expiratory pressure, SE: standard error, SOFA: sequential organ failure assessment scale, T0: at the time of diagnosis of AHRF, T24: at 24 hours of diagnosis of AHRF, VT: Tidal volume.

TABLE S7. Multicollinearity in the full model including 37 available variables within the first 24 hours of diagnosis of AHRF. In general, when the variance inflation factor (VIF) is higher than 5, variables are highly correlated; when the VIF is higher than 10, there is significant multicollinearity that needs to be corrected.

Variable	VIF
Age	1.69
Gender (female)	2.66
Arterial hypertension	1.52
Cardiac failure	1.22
Chronic renal failure	1.21
Diabetes	1.23
Immunocompromised	1.17
Morbid obesity	1.30
COPD	1.18
Neoplastic disease	1.12
Chronic liver failure	1.16
SOFA at T0	8.07
SOFA at T24	7.95
VT at T0	14.67
VT at T24	16.07
FiO_2 at T0	9.49
FIO_2 at T24	2.57
Respiratory rate at T0	24.71
Respiratory rate at T24	27.43
PEEP at T0	3.05
PEEP at T24	4.02
Plateau pressure at T0	5.85
Plateau pressure at T24	6
Driving pressure at T0	3.43
Driving pressure at T24	3.26
Minute ventilation at T0	30.90
Minute ventilation at T24	31.48
PaO_2 at T0	8.06
PaO_2 at T24	3.18
PaO_2/FiO_2 at T0	12.47
PaO_2/FiO_2 at T24	4.25
$PaCO_2$ at T0	2.02
$PaCO_2$ at T24	1.78
pH at T0	2.04
pH at T24	1.65
No. extrapulmonary OFs at T0	7.17
No. extrapulmonary OFs at T24	7.11

AHRF: acute hypoxemic respiratory failure, COPD: chronic obstructive pulmonary disease, OF: organ failures, PEEP: positive end-expiratory pressure, SOFA: sequential organ failure assessment scale, T0: at the time of diagnosis of AHRF, T24: at 24 hours of diagnosis of AHRF, VIF: variance inflation factor, VT: Tidal volume.

TABLE S8. Multivariate analysis and performance of a model of predicting ICU mortality (16-variable model) within 24 hours of diagnosis of AHRF using logistic regression analysis and minimizing the Akaike information criterion. This model reduced the number of variables from 37 to 16. Data are expressed as mean values of logistic coefficients.

Variable	b	SE	OR	95% CI	p-value
Intercept	8.22	10.76	3724.16	0 – 6.41E+12	0.445
Age	0.05	0.01	1.05	1.04 - 1.07	<0.001
Diabetes	0.32	0.21	1.38	0.91 - 2.08	0.125
Neoplastic disease	0.48	0.26	1.62	0.97 - 2.7	0.064
SOFA at T24	0.1	0.06	1.1	0.97 - 1.25	0.126
VT at T0	-0.24	0.16	0.78	0.57 - 1.07	0.129
VT at T24	-0.25	0.15	0.78	0.58 - 1.04	0.093
FiO_2 at T24	2.19	0.72	8.97	2.21 - 37.33	0.002
Respiratory rate at T0	-0.11	0.04	0.9	0.83 - 0.98	0.012
PEEP at T24	-0.23	0.04	0.8	0.73 - 0.86	<0.001
Plateau pressure at T24	0.27	0.02	1.31	1.26 - 1.38	<0.001
Minute ventilation at T0	0.18	0.09	1.2	1.01 - 1.43	0.042
PaO_2/FiO_2 at T0	0	0	1	1 - 1.01	0.096
$PaCO_2$ at T24	-0.03	0.01	0.97	0.95 - 0.99	0.016
pH at T0	1.84	1.04	6.28	0.84 - 49.06	0.076
pH at T24	-3.81	1.4	0.02	0 - 0.33	0.007
No. extrapulmonary OFs at T24	0.57	0.21	1.77	1.18 - 2.67	0.006
AIC	729.56				
BIC	811.201				
AUC ROC	0.893 (0.871 – 0.914)				

AHRF: acute hypoxemic respiratory failure, AIC: Akaike information criterion, AUC ROC: area under the receiving operating characteristic curve, BIC: Bayesian information criterion, CI: confidence intervals, OF: extrapulmonary organ failures included in the sequential organ failure assessment scale, OR: odds ratio, SE: standard error, SOFA: sequential organ failure assessment scale, T0: at the time of diagnosis/onset of AHRF, T24: at 24 hours of diagnosis of AHRF, VT: tidal volume.

TABLE S9. Multicollinearity in the 16-variable model by minimizing the Akaike information criterion (AIC) with available variables within the first 24 hours of diagnosis of AHRF. In general, when the variance inflation factor (VIF) is higher than 5, variables are highly correlated; when the VIF is higher than 10, there is significant multicollinearity that needs to be corrected.

Variable	VIF
Age	1.29
Diabetes	1.04
Neoplastic disease	1.03
SOFA at T24	4.75
VT at T0	3.05
VT at T24	2.63
FIO_2 at T24	1.55
Respiratory rate at T0	3.70
PEEP at T24	1.80
Plateau pressure at T24	1.70
Minute ventilation at T0	3.65
PaO_2/FiO_2 at T0	1.39
$PaCO_2$ at T24	1.41
pH at T0	1.33
pH at T24	1.44
No. extrapulmonary OFs at T24	4.45

AHR: acute hypoxemic respiratory failure, OF: extrapulmonary organ failures included in the sequential organ failure assessment scale, SOFA: sequential organ failure assessment scale, T0: at the time of diagnosis/onset of AHRF, T24: at 24 hours of diagnosis of AHRF, VT: tidal volume.

TABLE S10. Multicollinearity in the 6-variable model by minimizing the Bayesian information criterion (BIC) with available variables (n=6) within the first 24 hours of diagnosis of acute hypoxemic respiratory failure (AHRF). In general, when the variance inflation factor (VIF) is higher than 10, there is significant multicollinearity that needs to be corrected. In this model, there is a lack of collinearity.

Variable	VIF
Age	1.12
VT at T24	1.10
FIO_2 at T24	1.27
PEEP at T24	1.75
Plateau pressure at T24	1.53
No. extrapulmonary OFs at T24	1.04

AHRF: acute hypoxemic respiratory failure, OF: organ failures included in the sequential organ failure assessment scale, PEEP: positive end-expiratory pressure, T0: at the time of diagnosis/onset of AHRF, T24: at 24 hours after diagnosis of AHRF, VT: tidal volume.

TABLE S11: Distribution of 900 patients and 312 ICU deaths of the training/testing cohort, based on patient's age, PEEP, PaO_2, FiO_2, PaO_2/FiO_2 ratio, plateau pressure, number of extrapulmonary organ failures, and tidal volume (VT) at the time of diagnosis of acute hypoxemic respiratory failure (T0) and 24 hours later (T24). *Abbreviations: AHRF: acute hypoxemic respiratory failure; ICU: intensive care unit; N: total number; PaO_2/FiO_2: ratio of partial pressure of arterial oxygenation and inspired oxygen fraction; PBW: predicted body weight; PEEP: positive end-expiratory pressure.*

Variables of severity	At AHRF diagnosis (T0)		At 24 h later (T24)	
	Total N (%)	ICU deaths N (%)	Total N (%)	ICU deaths N (%)
**Age at ICU admission, years**				
≤50	183 (20.3)	40 (21.9)	-	-
51-70	408 (45.3)	131 (32.1)	-	-
>70	309 (34.3)	141 (45.6)	-	-
**p-value**	-	<0.0001	-	-
**PEEP, cmH_2O**				
<10	651 (72.3)	222 (34.1)	547 (60.8)	168 (30.7)
10-12	194 (21.6)	74 (38.1)	260 (28.9)	105 (40.4)
>12	55 (6.1)	16 (29.1)	93 (10.3)	39 (41.9)
**p-value**	-	0.3906	-	0.0078
**PaO_2, mmHg**				
≤75	215 (23.9)	76 (35.4)	111 (12.3)	51 (46.0)
>75	685 (76.1)	236 (34.5)	789 (87.7)	261 (33.1)
**p-value**	-	0.8696	-	0.0081
**FiO_2**				
<0.5	201 (22.3)	56 (27.9)	331 (36.8)	80 (24.2)
0.5-0.6	385 (42.8)	134 (34.8)	389 (43.2)	137 (35.2)
>0.6	314 (34.9)	122 (38.9)	180 (20.0)	95 (52.8)
**p-value**	-	0.0380	-	<0.0001
**PaO_2/FiO_2, mmHg**				
≤100	136 (15.1)	56 (41.2)	45 (5.0)	33 (73.3)
101-200	475 (52.8)	158 (33.3)	284 (31.6)	116(40.9)
>200	289 (32.1)	98 (33.9)	571 (63.4)	163 (28.6)
**p-value**	-	0.2198	-	<0.0001
**PaO_2/FiO_2, mmHg**				
<150	356 (39.6)	237 (38.5)	140 (15.6)	72 (51.4)
≥150	544 (60.4)	175 (32.2)	760 (84.4)	240 (31.6)
	-	0.0535	-	<0.0001
**Plateau pressure, cmH_2O**				
≤28	746 (82.9)	200 (26.8)	719 (79.9)	152 (21.1)
>28	154 (17.1)	112 (72.7)	181 (20.1)	160 (88.4)
**p-value**	-	<0.0001	-	0.0001
**Extrapulmonary organ failures**				
0	89 (9.9)	10 (11.2)	113 (12.6)	3 (2.7)
1-2	645 (71.7)	207 (32.1)	606 (67.3)	190 (31.4)
>2	166 (18.4)	95 (57.2)	181 (20.1)	119 (65.7)
**p-value**	-	<0.0001	-	<0.0001
**Tidal volume, ml/kg PBW**				
<4	6 (0.7)	5 (83.3)	8 (0.9)	6 (75.0)
4-6	156 (17.3)	58 (37.2)	156 (17.3)	62 (39.7)
>6-8	622 (69.1)	211 (33.9)	622 (69.1)	208 (33.4)
>8	116 (12.9)	38 (32.8)	114 (12.7)	36 (31.6)
**p-value**	-	0.0706	-	0.0383
**Total**	900	312 (34.7% of 900)	900	312 (34.7% of 900)

TABLE S12: Distribution by phases of total number and ICU deaths of the study population and the training/testing cohort

**Patients with data at T0 and T24 N=1193**			**Training/testing cohort N=900**	
Study's phases	***Total***	***ICU deaths** (n, %)*	***Total***	***ICU deaths** (n, %)*
Phase I	409	148 (36.2)	314	119 (37.9)
Phase II	378	124 (32.8)	273	91 (33.3)
Phase III	406	144 (35.5)	313	102 (32.6)
p-value	0.212	0.580	0.065	0.323

TABLE S13. Baseline characteristics and outcome data of 1193 patients with acute hypoxemic respiratory failure (AHRF) on mechanical ventilation distributed in the three phases of the study.

Variables	Phase I N = 409	Phase II N = 378	Phase III N = 406	p-value
Age, years, median (IQR)	65 (53-74)	65.5 (55-74)	65 (54-73)	
Age, years, mean ± SD	62.4± 15.1	63.2 ± 13.9	62.6 ± 14.1	0.722
Sex, n (%)				
Male	268 (65.5)	258 (68.3)	280 (69.0)	0.543
Female	141 (34.5)	120 (31.7)	126 (31.0)	
Etiology (reasons for invasive MV), *n* (%)				
Post-surgery	73 (17.8)	61 (16.1)	56 (13.8)	0.284
Stroke or coma	64 (15.6)	66 (17.5)	59 (14.5)	0.527
Pneumonia	42 (10.3)	50 (13.2)	75 (18.5)	0.003
Sepsis/Acute pancreatitis	56 (13.7)	43 (11.4)	47 (11.6)	0.541
Trauma	57 (13.9)	59 (15.6)	34 (8.4)	0.006
Cardiac arrest	38 (9.3)	28 (7.4)	42 (10.3)	0.352
Cardiac failure/fluid overload	20 (4.9)	20 (5.3)	19 (4.7)	0.923
Aspiration/Inhalation	18 (4.4)	17 (4.5)	12 (3.0)	0.454
Others	39 (9.5)	34 (9.0)	59 (14.5)	-
Unknown etiology	2 (0.5)	0 (0.0)	3 (0.7)	-
APACHE II score, mean ± SD	21.5 ± 7.8 §	20.7 ± 7.6 §	20.7 ± 8.0 §	0.244
SOFA score, mean ± SD	9.4 ± 3.5	8.7± 3.2	8.7± 3.4	0.004
FiO_2, mean ± SD	0.64 ± 0.22	0.60 ± 0.20	0.64 ± 0.22	0.011
PaO_2, mmHg, mean ± SD	99.5 ± 32.9	100.1 ± 35.9	97.0 ± 34.5	0.403
PaO_2/FiO_2, mmHg, mean ± SD	170 ± 64	177 ± 61	166 ± 65	0.131
$PaCO_2$, mmHg, mean ± SD	45.5 ±11.5	45.2 ±11.1	47.1 ±13.2	0.089
pH, mean ± SD	7.31 ± 0.11	7.32 ± 0.10	7.31 ± 0.11	0.324
VT, mL/kg PBW, mean ± SD	6.9 ± 1.1	6.9 ± 1.1	6.9 ± 1.0	1.0
Respiratory rate, cycles/min, mean ± SD	19.5 ± 4.4	19.8 ± 4.2	19.9 ± 4.5	0.398
Minute ventilation, L/min, mean ± SD	8.5 ± 2.1	8.6 ± 1.9	8.7 ± 2.1	0.376
PEEP, cmH_2O, mean ± SD	8 ± 3	8 ± 3	8 ± 3.0	1.0
Plateau pressure, cmH_2O, mean ± SD	22± 5	22 ± 5	22 ± 6	1.0
Driving pressure, cmH_2O, mean ± SD	14.6 ± 4.6	14.6 ± 4.7	14.1 ± 5.1	0.235
No. extrapulmonary OFs, mean ± SD	1.8 ± 1.1	1.7 ± 1.0	1.6 ± 1.0	0.054
Length of ICU stay, d, median (IQR)	10 (4 - 20)	12 (5 - 24)	11 (5 - 20)	0.368
Days from last day MV to ICU discharge, median (IQR)	2 (0-5)	2 (0-6)	2 (0-5)	0.284
All-cause ICU mortality, n (%)	148 (36.19)	124 (32.8)	144 (35.5)	0.580
All-cause hospital mortality, n (%)	175 (42.8)	150 (39.7)	164 (40.4)	0.617

APACHE: acute physiology and chronic health evaluation; CI: confidence intervals; d: days; FiO_2: fraction of inspired oxygen concentration; ICU: intensive care unit; IQR: interquartile range; MV: mechanical ventilation; OF: organ failure; PBW: predicted body weight; PEEP: positive end-expiratory pressure; SD: standard deviation; SOFA: sequential organ failure assessment scale; VT: tidal volume.

§ APACHE II was not reported at baseline in 7 patients on phase I, in 19 patients on phase II, and in 13 patients on phase III.

TABLE S14. Baseline features and outcome data of 900 mechanically ventilated patients (training/testing cohort) with acute hypoxemic respiratory failure distributed in the three phases of the parent study.

Variables	Phase I N=314	Phase II N=273	Phase III N=313	p-value
Age, years, median (IQR)	65 (52-74)	65 (55-73)	65 (53-73)	
Age, years, mean ± SD	62.1± 15.4	63.2 ± 13.7	62.5 ± 14.4	0.655
Sex, *n (%)*				
Male	206 (65.6)	189 (69.2)	212 (67.7)	0.641
Female	108 (34.4)	84 (30.8)	101 (32.3)	0.641
Etiology (reasons for invasive MV), *n* (%)				
Post-surgery	55 (17.5)	42 (15.4)	39 (12.5)	0.207
Stroke or coma	51 (16.2)	41 (15.0)	48 (15.3)	0.914
Pneumonia	31 (9.9)	42 (15.4)	60 (19.2)	0.004
Sepsis/Acute pancreatitis	43 (13.7)	34 (12.5)	36 (11.5)	0.708
Trauma	45 (14.3)	42 (15.4)	27 (8.6)	0.027
Cardiac arrest	28 (8.9)	20 (7.3)	31 (9.9)	0.543
Cardiac failure/fluid overload	13 (4.1)	14 (5.1)	17 (5.4)	0-737
Aspiration/Inhalation	16 (5.1)	12 (4.4)	9 (2.9)	0.361
Others	30 (9.6)	26 (9.5)	43 (13.7)	-
Unknown etiology	2 (0.6)	0 (0.0)	3 (1.0)	-
APACHE II score, mean ± SD	21.6 ± 7.9 §	20.8 ± 7.7 §	20.3 ± 7.9 §	0.113
SOFA score, mean ± SD	9.5 ± 3.5	8.8± 3.3	8.4± 3.2	<0.001
FiO_2, mean ± SD	0.65 ± 0.23	0.60 ± 0.20	0.64 ± 0.22	0.015
PaO_2, mmHg, mean ± SD	100 ± 34	100 ± 38	98 ± 35	0.725
PaO_2/FiO_2, mmHg, mean ± SD	170 ± 64	177 ± 61	166 ± 65	0.131
$PaCO_2$, mmHg, mean ± SD	45.0 ±11.1	45.1 ±11.6	46.9 ±13.1	0.087
pH, mean ± SD	7.32 ± 0.11	7.33 ± 0.10	7.32 ± 0.11	0.437
VT, mL/kg PBW, mean ± SD	6.9 ± 1.1	7.0 ± 1.1	6.9 ± 1.0	0.437
Respiratory rate, ventilator cycles/min, mean ± SD	19.3 ± 4.1	19.7 ± 4.1	19.7 ± 4.4	0.397
Minute ventilation, L/min, mean ± SD	8.5 ± 2.1	8.6 ± 1.9	8.7 ± 2.1	0.472
PEEP, cmH_2O, mean ± SD	8 ± 3	8 ± 3	8 ± 3	1.0
Plateau pressure, cmH_2O, mean ± SD	22.5 ± 5.3	22.5 ± 5.3	22.2 ± 5.9	0.746
Driving pressure, cmH_2O, mean ± SD	14.8 ± 4.6	14.8 ± 4.8	14.0 ± 5.3	0.067
0.054No. extrapulmonary OFs, mean ± SD	1.8 ± 1.1	1.7 ± 1.0	1.6 ± 1.0	0.054
Length of ICU stay, d, median (IQR)	10 (4 - 19)	12 (5 - 25)	10 (5 - 21)	0.347
Days from last day MV to ICU discharge, median (IQR)	1.5 (0-5)	2 (0-6)	2 (0-5)	0.395
All-cause ICU mortality, n (%: 95%CI)	119 (37.9)	91 (33.3)	102 (32.6)	0-323
All-cause hospital mortality, n (%: 95%CI)	141 (44.9)	110 (40.3)	118 (37.7)	0.179

APACHE: acute physiology and chronic health evaluation; C: confidence intervals; d: days; FiO_2: fraction of inspired oxygen concentration; ICU: intensive care unit; IQR: interquartile range; MV: mechanical ventilation; OF: organ failure; PBW: predicted body weight; PEEP: positive end-expiratory pressure; SD: standard deviation; SOFA: sequential organ failure assessment scale; VT: tidal volume.

§ APACHE II was not reported at baseline in 5 patients from phase I, 14 patients from phase II, and 11 patients from phase III.

TABLE S15. Comparison of analysis of performance using Multilayer Perception, Random Forest, Support Vector Machine, and Logistic Regression of the 6-variable model in 900 patients with acute hypoxemic respiratory failure (AHRF).

Methods	Model	N	AUC ROC (95%CI)	Sensitivity	Specificity	Accuracy	PPV	NPV
Multilayer Perceptron	6-variable	900	0.88 (0.84-0.93)	0.64	0.88	0.80	0.75	0.82
Random Forest	6-variable	900	0.86 (0.81-0.92)	0.65	0.86	0.79	0.72	0.82
Support Vector Machine	6-variable	900	0.85 (0.79-0.91)	0.63	0.89	0.80	0.75	0.82
Logistic regression	6-variable	900	0.88 (0.83-0.93)	0.86	0.76	0.79	0.69	0.91

AUC ROC: area under the receiver operating characteristic curve, CI: confidence intervals, NPV: negative predictive value, PPV: positive predictive value.

TABLE S16. Internal-external validation analysis of the 6-variable model (See Table S14 for details)**, representing the performance in a phase of the study developed in the other two phases** ("leave-one-phase-out")**.**

Model	N	Events	AUC ROC (95%CI)	Sensitivity	Specificity	Accuracy	PPV	NPV
Test on Phase I	314	119						
Multilayer Perceptron			0.84 (0.80-0.88)	0.67	0.75	0.72	0.62	0.79
Random Forest			0.83 (0.78-0.87)	0.70	0.79	0.75	0.67	0.81
Support Vector Machine			0.81 (0.76-0.85)	0.66	0.81	0.75	0.67	0.79
Logistic regression			0.84 (0.80-0.89)	0.89	0.61	0.72	0.58	0.90
Test on Phase II	273	91						
Multilayer Perceptron			0.90 (0.86-0.93)	0.52	0.96	0.81	0.87	0.80
Random Forest			0.88)0.83-0.92)	0.67	0.90	0.82	0.77	0.85
Support Vector Machine			0.86 (0.82-0.91)	0.62	0.90	0.80	0.75	0.82
Logistic regression			0.89 (0.85-0.93)	0.88	0.76	0.80	0.65	0.93
Test on Phase III	313	102						
Multilayer Perceptron			0.91 (0.88-0.94)	0.62	0.96	0.85	0.88	0.84
Random Forest			0.91 (0.87-0.94)	0.67	0.91	0.83	0.79	0.85
Support Vector Machine			0.86 (0.81-0.90)	0.62	0.94	0.84	0.84	0.84
Logistic regression			0.89 (0.85-0.93)	0.78	0.84	0.82	0.71	0.89
	900	312						

AUC ROC: area under the receiver operating characteristic curve, CI: confidence intervals, NPV: negative predictive value, PPV: positive predictive value.

TABLE S17. Validation of the 6-variable model for ICU outcome in an independent (unseen) cohort of 293 patients with acute hypoxemic respiratory failure. NOTE: We used the 900-patient population cohort as the training cohort and the new (unseen) 293 patients as the testing cohort.

Methods	Model	AUC ROC (95%CI)	Sensitivity	Specificity	Accuracy	PPV	NPV
Multilayer Perceptron	6-variable	0.83 (0.78-0.88)	0.49	0.95	0.79	0.85	0.77
Ransom Forest	6-variable	0.78 (0.73-0.84)	0.64	0.78	0.73	0.61	0.80
Support Vector Machine	6-variable	0.78 (0.72-0.84)	0.62	0.83	0.75	0.67	0.80
Logistic Regression	6-variable	0.82 (0.77-0.87)	0.67	0.83	0.77	0.68	0.82

AUC ROC: area under the receiver operating characteristic curve, CI: confidence intervals, NPV: negative predictive value, PPV: positive predictive value.

FIGURE S1. Time line for the early prediction model on ICU mortality in patients with acute hypoxemic respiratory failure (AHRF). At the patient's time of eligibility (i.e. when patients develop AHRF), the patient's risk of future outcome at ICU discharge (alive or death) was predicted using the first 24 hours of data.

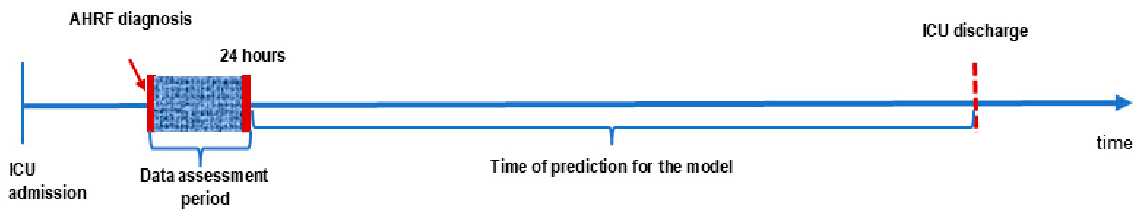

FIGURE S2. Steps for the prediction model development in 1193 patients with hypoxemic respiratory failure (AHRF). We performed logistic regression analysis, and internal–external validation. For internal validation we used all 900 patients, and then by leaving each of the three parent phases out once, performance was estimated in each left-out phase. Revalidation was conducted by testing the model in the 293 unseen AHRF patients.

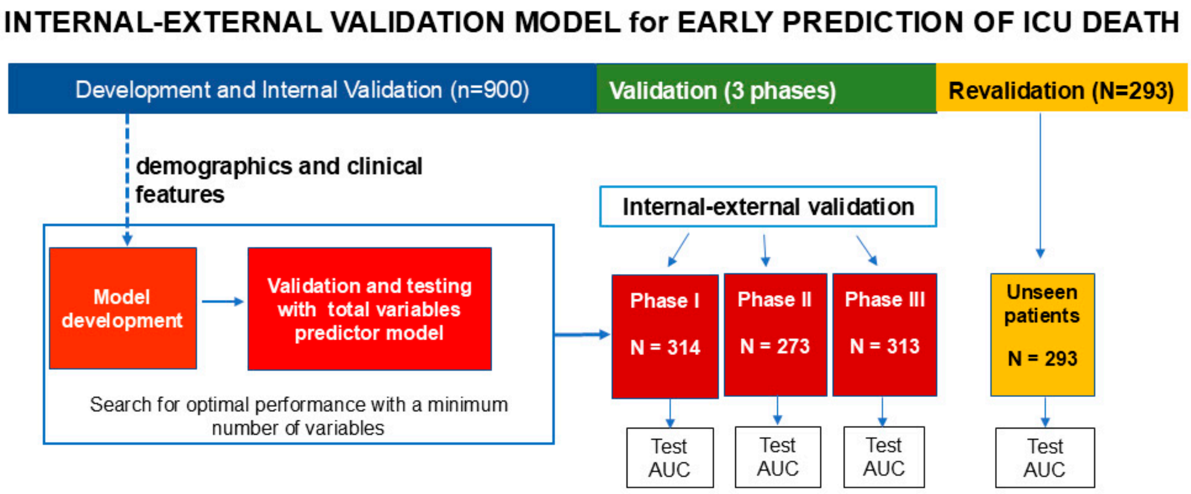

FIGURE S3. Correlation matrices at T0 and T24 associated with death in the ICU of 25 variables within 24 hours after diagnosis of acute hypoxemic respiratory failure (AHRF). Blue represents positive correlation and red represents negative correlation. The area of the pie chart represents the specific value of correlation coefficients.

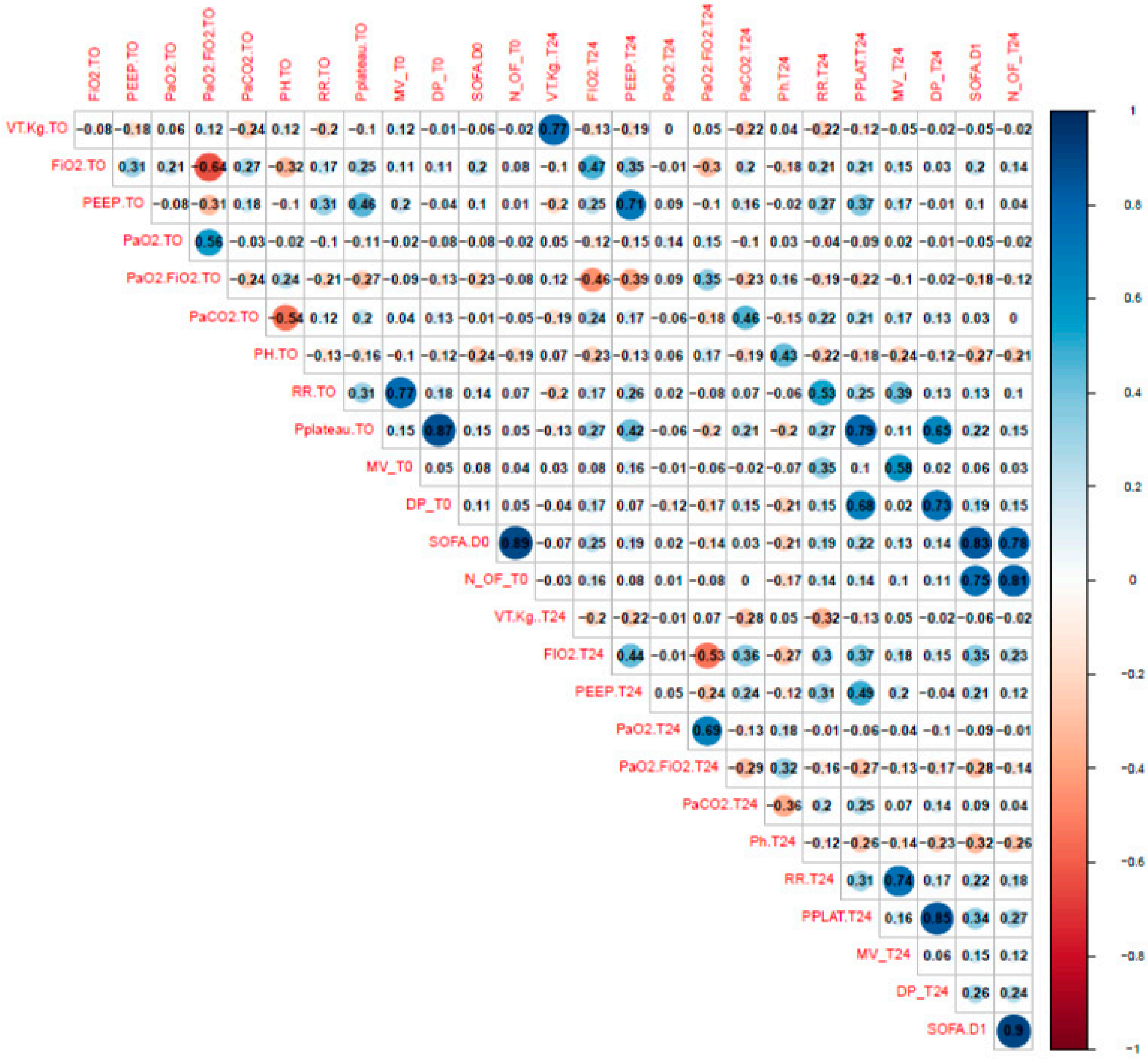

FIGURE S4. Principal component analysis (PCA) of predictors of death in the ICU in patients with acute hypoxemic respiratory failure (AHRF). Cluster of surviving ICU patients is marked in red, and cluster of dead ICU patients is marked in green. Although there is overlapping between the clusters, the two clusters differed more at T24 (Dim1 + Dim2 = 35.1% + 19.8% = 54.9%).

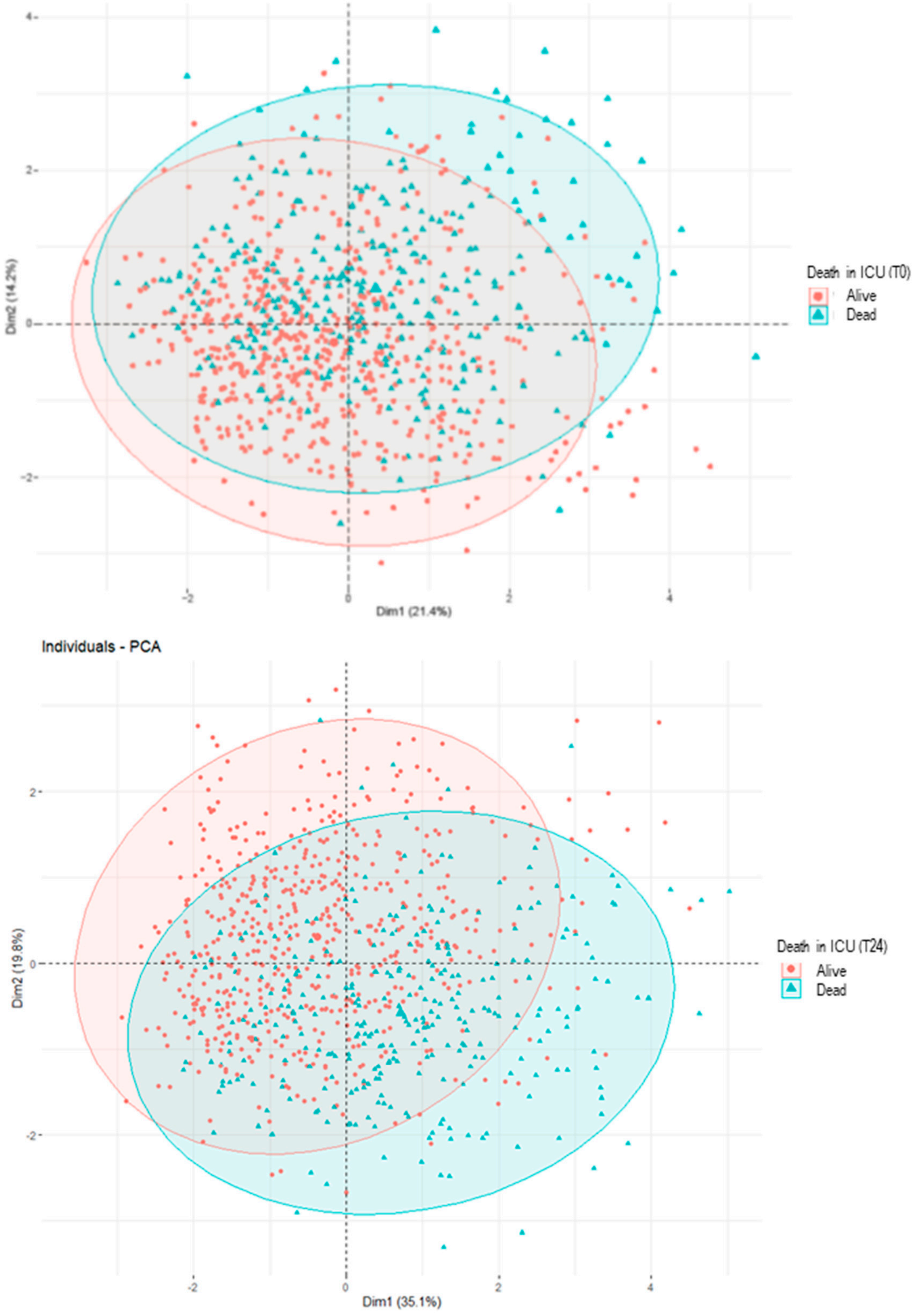

FIGURE S5. Variable importance of variables in the 6-variable model in terms of principal component analysis (PCA) of predictors of death in ICU in patients with acute hypoxemic respiratory failure (AHRF). *Abbreviations: Contrib, Contributions by color of the variable importance; Dim, Dimensions of the principal component analysis; FiO$_2$, fraction of inspired oxygen; No. OFs, number of extrapulmonary organ failures; OF, organ failure; PEEP, positive end-expiratory pressure; Pplat, inspiratory plateau pressure; T0: at the time of diagnosis of AHRF; T24: at 24 h after AHRF diagnosis; VT, tidal volume.*

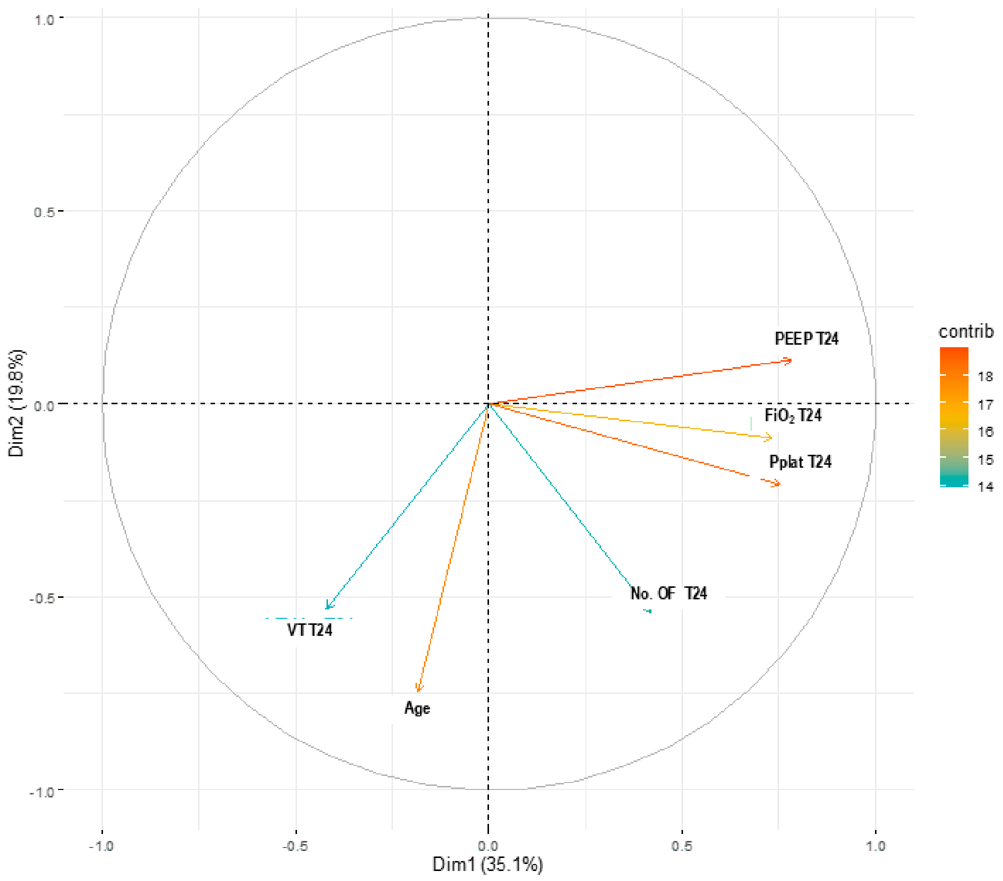

FIGURE S6. Calibration plots of the multilayer perceptron-based (A), random forest-based (B), support vector machine-based (C), and logistic regression based (D) ICU mortality prediction model for the 6-variable model in patients with hypoxemic respiratory failure. The intercept relates to calibration-in-the large, which compares mean observed with mean predicted risks. The calibration slope reflects the coefficient of the calibration plot. The c-statistics indicates the discriminative ability.

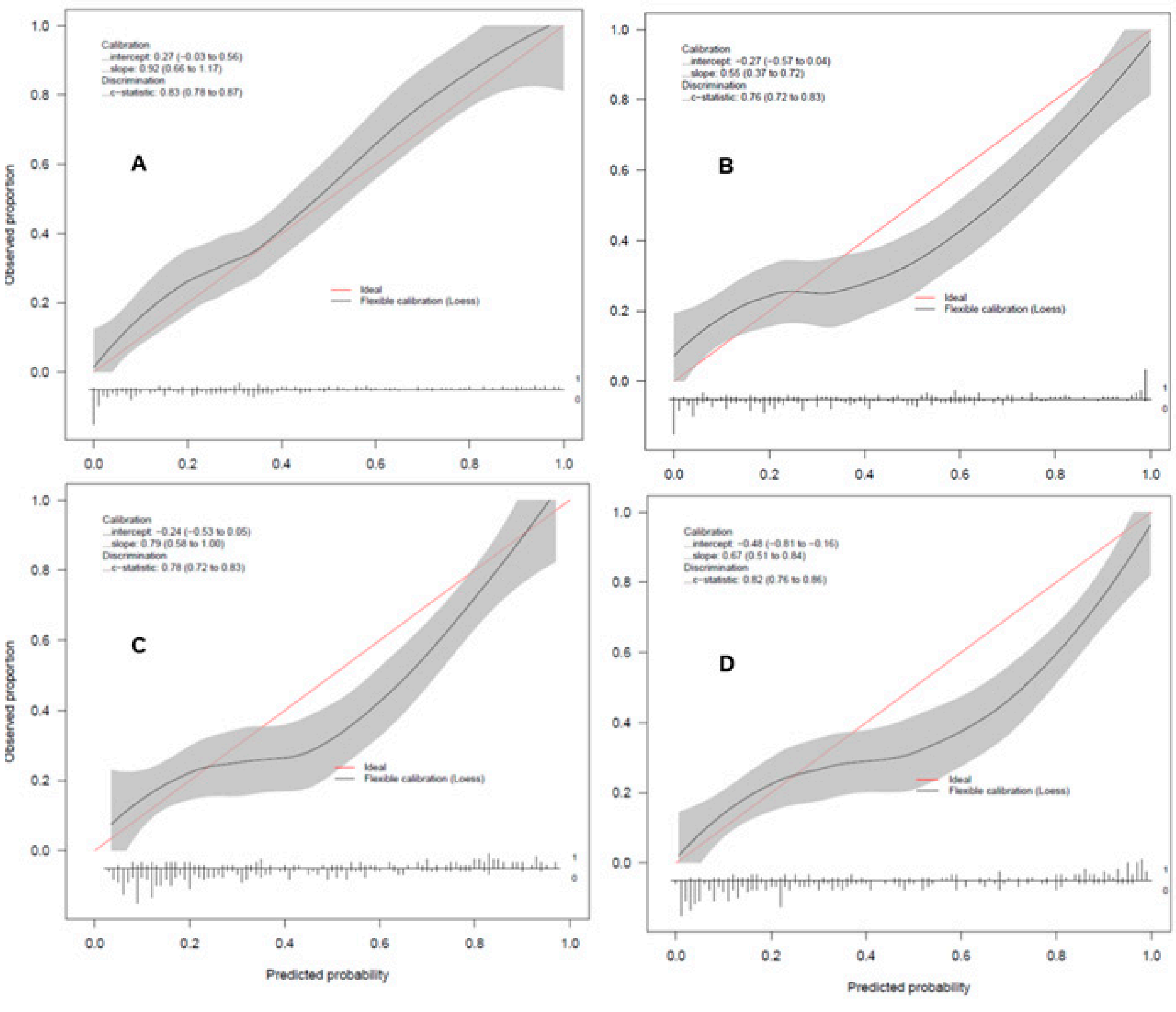

SUPPLEMENTARY REFERENCES

1. Villar J, Mora-Ordoñez JM, Soler JA, Mosteiro F, Vidal A, Ambrós A, Fernández L, Murcia I, Civantos B, Romera MA, et al. The PANDORA study: prevalence and outcome of acute hypoxemic respiratory failure in the pre-Covid-19 era. Crit Care Expl 2022; 4:e0684.
2. Parsa-Parsi RW. The International Code of Medical Ethics of the World Medical Association. JAMA 2022. doi: 10.1001/jama.2022.19697. Online ahead of print (PMID 36227623).
3. Collins GS, Reitsma JB, Altman DG, Mooms KGM. Transparent reporting of a multivariable prediction model for individual prognosis or diagnosis (TRIPOD): The TRIPOD statement. J Clin Epidemiol 2015; 68:112-121.
4. Leisman DE, Harhay MO, Lederer DJ, Abramson M, Adjei AA, Bakker J, Ballas ZK, Barreiro E, Bell SC, Bellomo R, et al. Development and reporting of prediction models: Guidance for authors from editors of respiratory, sleep, and critical care journals. Crit Care Med 2020; 48:623-633.
5. Villar J, González-Martín JM, Hernández-González J, Armengol MA, Fernández C, Martín-Rodríguez C, Mosteiro F, Martínez D, Sánchez-Ballesteros J, Ferrando C, et al. Predicting ICU mortality in acute respiratory distress syndrome patients using machine learning: the predicting outcome and stratification of severity in ARDS (POSTCARDS) study. Crit Care Med 2023; 51:1638-1649.
6. Steyerberg EW, Harrel FE: Prediction models need appropriate internal, internal-external, and external validation. J Clin Epidemiol 2016; 69:245-247.
7. Steyerberg EW, Harrel FE, Borsboom GJJM, Eijkemans MJCR, Vergouwe Y, Habbema JDF: Internal validation of predictive models: efficiency of some procedures for logistic regression analysis. J Clin Epidemiol 2011; 54:774-781.
8. Knaus WA, Draper EA, Wagner DP, Zimmerman JE. APACHE II: a severity of disease classification system. Crit Care Med 1985; 13:818-829.
9. Vincent JL, de Mendonça A, Cantraine F, Moreno R, Takala J, Suter PM, Sprung CL, Colardyn F, Blecher S. Use of the SOFA score to assess the incidence of organ dysfunction/failure in intensive care units: results of a multicenter, prospective study. Working group on "sepsis-related problems" of the European Society of Intensive Care Medicine. Crit Care Med 1998; 26:1793-1800.

10. Singer M, Deutschman CS, Seymour CW, Shankar-Hari M, Annane D, Bauer M, Bellomo R, Bernard GR, Chiche JD, Coopersmith CM, et al. The third international consensus definitions for sepsis and septic shock (sepsis-3). JAMA 2016; 315:801-810.
11. Acute Respiratory Distress Syndrome Network. Ventilation with lower tidal volumes as compared with traditional tidal volumes for acute lung injury and the acute respiratory distress syndrome. N Engl J Med 2000; 342:1301-1308.
12. Kacmarek RM. Noninvasive respiratory support for postextubation respiratory failure. Respir Care 2019; 64:658.678.
13. Kacmarek RM, Villar J, Sulemanji D, Montiel R, Ferrando C, Blanco J, et al. Open lung approach for the acute respiratory distress syndrome: A pilot, randomized controlled trial. Crit Care Med 2016; 44:32-42.
14. Eke G, Bloos F, Wilson DC, Meybohm P, SepNet Critical Care Trials Group. Identification of developing multiple organ failure in sepsis patients with low or moderate SOFA scores. Crit Care 2018; 22:147.
15. Villar J, Martínez D, Mosteiro F, Ambrós A, Añón JM, Ferrando C, Soler JA, Montiel R, Vidal A, Conesa-Cayuela LA, Blanco J; Stratification and Outcome of Acute Respiratory Distress Syndrome (STANDARDS) Network. Crit Care Med 2018; 46:892-899.
16. Ranieri VM, Rubenfeld GD, Thompsom BT, Ferguson ND, Caldwell E, Fan E, Camporota L, Slutsky AS. Acute respiratory distress syndrome. The berlin definition. JAMA 2012; 307:2526-2533.
17. Mandrekar JN. Receiver operating characteristic curve in diagnostic test assessment. J Thorac Oncol 2010; 5:1315-1316.
18. Villar J, González-Martín JM, Ambrós A, Mosteiro F, Martínez D, Fernández L, Soler JA, Parra L, Solano R, Soro M; Spanish Initiative for Epidemiology, Stratification and Therapies of ARDS (SIESTA) Network. Stratification for Identification of Prognostic Categories In the Acute RESpiratory Distress Syndrome (SPIRES) Score. Crit Care Med. 2021; 49:e920-e930.
19. Rauf A, Sachdev A, Venkataraman ST, Dinand V. Dynamic Airway Driving Pressure and Outcomes in Children With Acute Hypoxemic Respiratory Failure. Respir Care. 2021; 66:403-409.
20. Scrucca L. GA: a package for genetic algorithms in R. J Statist Softw 2013; 53:1-37.

21. Vrieze SI. Model selection and psychological theory: a discussion of the differences between the Akaike information criterion (AIC) and the Bayesian information criterion (BIC). Psychol Methods 2012; 17:228-243.
22. Kim JH. Multicollinearity and misleading statistical results. Korean J Anesthesiol 2019; 72:558-569.
23. Wang X, Meng L, Zhang J, Zhao Z, Zou L, Jia Z, Han X, Zhao L, Song M, Zong J, et al. Identification of ferroptosis-related molecular clusters and genes for diabetic osteoporosis based on the machine learning. Front Endocrinol 2023; 14:1189513.
24. Wang X, Meng L, Zhang J, Zhao Z, Zou L, Jia Z, Han X, Zhao L, Song M, Zong J, et al. Identification of ferroptosis-related molecular clusters and genes for diabetic osteoporosis based on the machine learning. Front Endocrinol 2023; 14:1189513.
25. Jolliffe IT, Cadima J. Principal components analysis: a review and recent developments. Philos Trans A Math Phys Eng Sci 2016; 374:20150202.
26. Rashid M, Ramakrishnan M, Pulikkel V, Nandish S, Nair S, Shanbhag V, Thunga G. Artificial intelligence in acute respiratory distress syndrome: a systematic review. Artif Intell Med 2022; 131:102361.
27. Sayed M, Riaño D, Villar J. Predicting duration of mechanical ventilation in acute respiratory distress syndrome using supervised machine learning. J Clin Med 2021; 10:3824.
28. Boulesteix AL, Janitza S, Kruppa J, König IR. Overview of random forest methodology and practical guidance with emphasis on computational biology and bioinformatics. Wiley Interdiscip. Rev Data Min Knowl Discov 2012; 2:493–507.
29. Khalilzad Z, Hasasneh A, Tadj C. Newborn cry-based diagnostic system to distinguish between sepsis and respiratory distress syndrome using combined acoustic features. Diagnostics (Basel) 2022; 12:2802.
30. Jeon ET, Lee HJ, Park TY, Jin KN, Ryu B, Lee HW, Kim DH. Machine learning-based prediction of in-ICU mortality in pneumonia patients. Sci Rep 2023; 13:11527.
31. Steyerberg EW, Harrel FE, Bosbsboom GJM, Eijkemans MJ, Vergouwe Y, Habbema JD. Internal validation of prediction models: efficiency of some procedures for logistic regression analysis. J Clin Epidemiol 2001; 54:774-781.
32. Saxena A, Mathur N, Pathak P, Tiwari P, Mathur SK. Machine learning model based on insulin resistance metagenes underpins genetic basis of type 2 diabetes. Biomolecules 2023; 13:432.

33. Ioannidis JPA. The proposal to lower P value thresholds to 0.005. JAMA 2018; 319:1429-1430.

34. Martínez-Taboada F, Redondo JI. The SIESTA (SEAAV Integrated evaluation sedation tool for anaesthesia) project: Initial development of a multifactorial sedation assessment tool for dogs. PLoS One 2020; 15: e0230799.

35. Van Calster B, Nieboer D, Vergouwe Y, De Cock B, Pencina MJ, Steyerberg EW. A calibration hierarchy for risk models was defined: from utopia to empirical data. J Clin Epidemiol 2016; 74:167-176.

APPENDIX S1. List of centers and investigators involved in the MEMORIAL study

Centers	Department/Hospital	Address and city/province	Investigators
1	Intensive Care Unit,<br>Hospital Universitario de La Paz	Paseo de la Castellana 261<br>28046 Madrid, Spain	José M. Añón<br>Belén Civantos<br>Mónica Hernández
2	Intensive Care Unit,<br>Hospital Virgen de La Luz	Hermandad Donantes de Sangre<br>1 16002 Cuenca, Spain	Elena González<br>Rosario Solano
3	Intensive Care Unit,<br>Complejo Asistencial Universitario de León	Altos de Nava s/n<br>24001 León, Spain	Ana M. Domínguez-Berrot<br>F. Javier Díaz-Domínguez<br>Raúl I. González Luengo<br>Myriam González-Vaquero
4	Post-Surgical Care Unit,<br>Hospital Clínico Universitario de Valencia	Blasco Ibáñez 17<br>46010 Valencia, Spain	Carlos Ferrando*<br>Blanca Arocas<br>Marina Soro<br>Andrea Gutiérrez<br>Gerardo Aguilar
5	Intensive Care Unit,<br>Hospital Universitario Río Hortega	Dulzaina 2<br>47012 Valladolid, Spain	Lorena Fernández<br>Jesús Sánchez-Ballesteros<br>Arturo Muriel<br>Pablo Blanco-Schweizer<br>José Ángel de Ayala<br>Jesús Blanco
6	Intensive Care Unit,<br>Hospital Clínico Universitario de Valladolid	Avda. Ramón y Cajal 3<br>47003 Valladolid, Spain	Leonor Nogales<br>David Andaluz<br>Laura Parra
7	Intensive Care Unit,<br>Hospital Universitario Virgen de Arrixaca	Ctra. Madrid-Cartagena s/n<br>30120 El Palmar, Murcia, Spain	Juan A. Soler<br>Domingo Martínez<br>Ana M. del Saz-Ortiz<br>Luís A. Conesa-Cayuela

8	Intensive Care Unit,<br>Hospital General Universitario de Ciudad Real	Obispo Rafael Torija s/n<br>13005 Ciudad Real, Spain	Alfonso Ambrós<br>Rafael del Campo<br>Carmen Martínez-Rodriguez<br>Ana Bueno-González<br>Carmen Hornos-López
9	Intensive Care Unit,<br>Hospital Universitario NS de Candelaria	Ctra. Del Rosario 145<br>38010 Santa Cruz de Tenerife, Spain	Raquel Montiel<br>Dácil Parrilla<br>Eduardo Peinado<br>Lina Pérez-Méndez
10	Intensive Care Unit,<br>Hospital Universitario 12 de Octubre	Avda. de Córdoba s/n<br>28041 Madrid, Spain	Isidro Prieto<br>Mario Chico
11	Intensive Care Unit,<br>Hospital Universitario Puerta de Hierro	Manuel de Falla 1<br>28222 Majadahonda, Madrid, Spain	Miguel A. Romera<br>Carlos Chamorro-Jambrina
12	Intensive Care Unit,<br>Hospital Universitario Regional	*Carlos Haya* s/n<br>29010 *Málaga, Spain*	Juan M. Mora-Ordoñez<br>J. F. Martínez-Carmona<br>Álvaro Valverde-Montoro<br>Victoria Olea-Jiménez
13	Intensive Care Unit,<br>Hospital NS del Prado	Ctra Madrid Km 114, 45600<br>Talavera de la Reina, Toledo, Spain	Paco Alba<br>Ruth Corpas
14	Intensive Care Unit,<br>Hospital Universitario de A Coruña	As Xubias 84<br>15006 A Coruña, Spain	Fernando Mosteiro<br>Lidia Pita-García<br>Ana M. Díaz-Lamas
15	Intensive Care Unit,<br>Hospital El Bierzo, Ponferrada	Médicos sin Fronteras 7<br>24404 Ponferrada, León, Spain	Eleuterio Merayo<br>Chanel Martínez<br>Ángeles de Célis-Álvarez
16	Intensive Care Unit,<br>Hospital La Mancha Centro	Avda. Constitución 3, 13600<br>Alcázar de S. Juan, Ciudad Real, Spain	Carmen Martín-Delgado

17	Post-operative Care Unit,<br>Hospital Universitario Río Hortega	Dulzaina 2<br>47012 Valladolid, Spain	César Aldecoa<br>Alba Pérez<br>Jesús Rico-Feijoo
18	Post-operative Care Unit,<br>Hospital Universitrario Ramón y Cajal	Ctra. Colmenar Viejo Km 9.1<br>28034 Madrid, Spain	David Pestaña<br>Adrián Mira<br>Pilar Cobeta
19	Intensive Care Unit,<br>Hospital Universitario Mutua Terrassa	Plaça del Dr. Robert 5<br>08221 Terrassa, Barcelona,<br>Spain	María del Mar Fernández
20	Intensive Care Unit,<br>Hospital Virgen de la Concha	Avda. Requejo 35<br>49022 Zamora, Spain	Concepción Tarancón<br>Silvia Cortés-Díaz
21	Intensive Care Unit,<br>Hospital Fundación Jiménez Díaz	Avda. Reyes Católicos 2<br>28040 Madrid, Spain	Anxela Vidal<br>Denis Robaglia<br>César Pérez
22	Intensive Care Unit,<br>Hospital Universitario de Albacete	Hermanos Falcó 37<br>02006 Albacete, Spain	Isabel Murcia<br>Ángel E. Pereyra-Pache
23	Research Unit,<br>Hospital Universitario Dr. Negrín	Barranco de la Ballena s/n.<br>35019 Las Palmas de Gran<br>Canaria, Spain	Jesús Villar<br>Jesús M. González-Martín<br>Cristina Fernández<br>Estrella Gómez-Bentolila<br>(not enrolling patients)
24	Department of Biomedical Data<br>Sciences, Leiden University Meical<br>Center	Albinusdreef 2, 2333 ZA Leiden,<br>The Netherlands	Ewout W. Steyerberg<br>(not enrolling patients)
25	Department of Anaesthesia, Intensive<br>Care and Pain Medicine.	Cardiff University<br>Cardiff CF14 4XN,<br>United Kingdom	Tamas Szakmany<br>(not enrolling patients)

NOTE: One center (unlisted) abandoned the study during the first 2-month period because of absence of its local investigator.

(*) current affiliation: Department of Anesthesia and Critical Care, Hospital Clinic, Barcelona, Spain.
